# Identification of Pedigree Relationship from Genome Sharing

**DOI:** 10.1534/g3.113.007500

**Published:** 2013-09-01

**Authors:** William G. Hill, Ian M. S. White

**Affiliations:** Institute of Evolutionary Biology, School of Biological Sciences, University of Edinburgh, Edinburgh, EH9 3JT, United Kingdom

**Keywords:** relationship, identity-by-descent, genomic identity, likelihood

## Abstract

Determination of degree of relationship traditionally has been undertaken using genotypic data on individual loci, typically assumed to be independent. With dense marker data as now available, it is possible to identify the regions of the genome shared identical by descent (ibd). This information can be used to determine pedigree relationship (**R**), *e.g.*, cousins *vs.* second cousins, and also to distinguish pedigrees that have the same Wright’s relationship (*R*) such as half-sibs and uncle–nephew. We use simulation to investigate the accuracy with which pedigree relationship can be inferred from genome sharing for uniparental relatives (a common ancestor on only one side of their pedigree), specifically the number, position (whether at chromosome ends), and length of shared regions ibd on each chromosome. Moments of the distribution of the likelihood ratio (including its expectation, the Kullback-Leibler distance) for alternative relationships are estimated for model human genomes, with the ratio of the mean to the SD of the likelihood ratio providing a useful reference point. Two relationships differing in *R* can be readily distinguished provided at least one has high *R*, *e.g.*, approximately 98.5% correct assignment of cousins and half-cousins, but only approximately 75% for second cousins once removed and third cousins. Two relationships with the same *R* can be distinguished only if *R* is high, *e.g.*, half-sibs and uncle–nephew, with probability of correct assignment being approximately 5/6.

Relatives carry individual genes and also genomic regions identical by descent (ibd). In many situations in human, natural, or agricultural populations, it is important to identify relatives and, if possible, degree of relationship using this information. Traditionally, methods of identifying relatives have used information regarding identity in state (ibs) of individual genes (Weir 1996), with increasingly dense markers enabling increasingly high precision, using methods such as CERVUS (Marshall *et al.* 1998), components of PLINK (Purcell *et al.* 2007), or COANCESTRY (Wang 2011).

Traditionally, establishing relationships does not use information regarding location in the genome, and statistical properties are often based on assuming unlinked markers. Linkage information can be incorporated, however, by using the linkage map and taking into account the Markovian nature of the ibd process underlying the genotypes of relatives at linked loci (Epstein *et al.* 2000; McPeek and Sun 2000; Kyriazopoulou-Panagiotopoulou *et al.* 2011) using methods such as RELPAIR (Epstein *et al.* 2000). Regions of the genome that are shared ibd can be established using identity in state (*e.g.*, Abecasis *et al.* 2002; Roberson and Pevsner 2009) using programs such as MERLIN (Abecasis *et al.* 2002).

Alternatively, distantly related individuals can be identified from multilocus sharing of even quite small regions of the genome (Browning and Browning 2011, 2012, 2013). If it is known that two individuals are related, then the allelic information adds little on regions already clearly shared ibd as determined by common sequence (except perhaps on ibs of two very-low-frequency genes). Further, the use of information on shared regions rather than just individual loci allows, at least in principle, discrimination between relationships with the same Wright’s relationship *R* but different pedigree **R**, *e.g.*, uncle–nephew and half-sib, both of which have *R* = 0.25. *R* used here is strictly Wright’s *numerator* relationship, which equals twice the kinship (coancestry), but it is the same as Wright’s relationship in the absence of inbreeding. **R** defines the pedigree (Table 1). Further, the actual proportion of the genome shared can, by chance, be higher by more distant (*e.g.*, second cousins: *R* = 1/32) than closer relatives (*e.g.*, first cousins once removed: *R* = 1/16). The proportion of overlap of the distribution of actual relationship increases as the relationship of each of the pairs becomes more distant (*e.g.*, *R* = 1/64 *vs.* 1/32) (Hill and Weir 2011), further increasing the problem of determining the pedigree **R**.

**Table 1:** Pedigree relationships (R), Wright’s coefficient of relationship (*R*), and abbreviations used

Relationship *R*	Full-sib family-based **R**		Half-sib family-based **R**		Lineal **R**	
1/4	Uncle–nephew*^a^*	UN	Half-sibs	HS	Grandparent–grandoffspring	GPO
1/8	Great-uncle–great-nephew	GUGN	Half-uncle–nephew	HUN	Great-grandparent–great-grandoffspring	GGPO
1/8	Full cousins	C				
1/16	Cousins once removed	C1R	Half-cousins	HC	GtGtgrandparent–	G3PO
					GtGtgrandoffspring	
1/16			Half-great-uncle–great-nephew*^b^*			
1/32	Second cousins	2C	Half-cousins once removed	HC1R	GtGtGtgrandparent–	G4PO
					GtGtGtgrandoffspring	
1/64	Second cousins once removed	2C1R	Half second cousins	H2C	Further generation as above	G5PO
1/128	Third cousins	3C	Half second cousins once removed	H2C1R	Further generation as above	G6PO

UN, uncle–nephew; HS, half-sib; GPO, grandparent–grandoffspring; GUGN, great-uncle–great-nephew; HUN, half-uncle–nephew; GGPO, great-grandparent–great-grandoffspring; C, cousin; C1R, cousin once removed; HC, half-cousin; Gt, great; G3PO, great-great-grandparent–great-great-grandoffspring; 2C, second cousin; HC1R, half-cousin once removed; G4PO, great-great-great-grandparent–great-great-great-grandoffspring; 2C1R, second cousin once removed; H2C, half second cousin; G5PO, great-great-great-great-grandparent–great-great-great-great-grandoffspring; 3C, third cousin; H2C1R, half second cousin once removed; G6PO, great-great-great-great-great-grandparent–great-great-great-great-great-grandoffspring.

a Including uncle/aunt–nephew/niece.

b Relationship not included in subsequent Tables as the distribution is identical to that for half-cousins.

Similarly, other relationships with the same *R* and family base but different pedigree are not analyzed, *e.g.*, (full) cousins twice removed ≡ second cousins (*R* = 1/32), and half-cousins twice removed ≡ half second cousins (*R =* 1/64).

The pedigree relationship may be needed in a number of situations. The estate of an individual who dies intestate may by law have to be divided among his or her closest relatives. Courts would assume this to be defined by pedigree. Another situation would be in identification of individuals in forensic cases, for example, in identifying a body or a body part in a disaster zone, or in familial searching for relatives of an offender already in the database (Rohlfs *et al.* 2012). In studies of natural populations, pedigree construction is an important component in determining breeding structure and estimation of genetic parameters (Blouin 2003; Pemberton 2008).

Detection of genomic regions for which there is biparental sharing, *i.e.*, individuals with ibd genotype at each diploid locus due to relationship through both parents (*e.g.*, full-sibs or double first cousins), is quite straightforward because there is ibs at each locus in the region. We consider here only the much more common situation of uniparental sharing, in which case *R* is half the probability relatives share one allele ibd at a locus, or half the expected proportion of uniparental genome shared.

Therefore, a quantitative description of the number, position, and length of shared regions is all the information we can have about relationship of a pair of individuals in the absence of pedigree information, and this sets an upper limit to what we can detect. Our objective is to find what this limit is for different alternative pedigrees. Therefore, as a reference point, we work on the premise that we have precise estimates of these quantities but later consider this assumption. We also assume there are no confounding factors, such as inbreeding of the common ancestor or relationships among other ancestors of the pair. Data on gene frequencies and genotypes at individual loci then add no further information.

We focus on identifying specified pedigree relationship from actual or realized genomic sharing, for example, whether a pair of individuals are related either as second cousins or as cousins once removed, in each case assuming there is uniparental sharing. Such comparisons can be undertaken based on a likelihood ratio, although the appropriate test or discrimination depends on the questions to be answered, such as the following. Which of two or more alternative relationships is the most probable? How sure are we? What relationships can we exclude?

The variation in the total length can be computed (Hill and Weir 2011) and there are also various methods and approximations for computing the numbers and distribution of the lengths of shared regions (Fisher 1954; Donnelly 1983; Stam 1980). Recently, Huff *et al.* (2011) have proposed methods to identify whether pairs of individuals taken from the population are related more closely, *e.g.*, as second cousins, than background relationship among all population members from distant relationships in a finite closed population.

There is no theory available that enables prediction of the numbers and distribution of shared segments exactly for arbitrary relationships. Therefore, we use simulation to generate the required probability distributions. There are, however, approximations for some of these distributions available: for example, Huff *et al.* (2011) assumed a Poisson distribution of number and exponential distribution of shared regions (*i.e.*, independence), and we also investigated their accuracy. We conclude with a discussion on inference. The primary objective was to set the theoretical framework and compute what can be achieved rather than focus on applications *per se*.

## Materials and Methods

### Simulation

The simulation program was used previously to check theoretical results for the variance of the length of shared regions on a chromosome (Hill and Weir 2011), which in turn provided a check on the program itself. Simulations were undertaken for a single chromosome, for example, of length *l* Morgans, in each independent replicate. There was assumed to be a uniform recombination rate and no interference, *i.e.*, corresponding to a Haldane mapping function. The number of recombination events was sampled from a Poisson distribution and their positions were sampled as real valued numbers independently from the uniform distribution. All regions of ancestral chromosomes were labeled by the same integer value, *e.g.*, 1, 2, and so on. Hence, a chromosome of a descendant was defined by the position (*π*) of each of the *n* − 1 recombination events, *e.g.*, 0 = *π*_0_ < *π*_1_, *π*_2_, …, *π_n_*_−1_ < *π_n_* = 1, defining the *n* chromosomal regions labeled *h*_1_, …, *h_n_*. Then, for example, *n* = 4, *π*_1_ = 0.1256, *π*_2_ = 0.5701, *π*_3_ = 0.9012, and *h*_1_ = 1, *h*_2_ = 2¸ *h*_3_ = 1, *h*_4_ = 3 denote a chromosome for which the first region (from 0.0 to 0.1256) and third region (from 0.5701 to 0.9012) were derived from ancestor 1, the second was derived from ancestor 2, and the third was derived from ancestor 3, and thus ibd for that genomic region with these respective ancestors. This does not imply that the parent has a chromosome with exactly that haplotype, but that a gamete could be formed from it that does, *i.e.*, the shared region may span grandpaternal and grandmaternal origins. Hence, for a second descendent of the same individuals with, for example, *n* = 2 and *π*_1_ = 0.3659, *h*_1_ = 2, *h*_2_ =1, there is sharing between the two descendents in two regions, between 0.1256 and 0.3659 from ancestor 2 and between 0.5701 and 0.9012 from ancestor 1, *i.e.*, internal regions of length 0.2403 and 0.3311, respectively, with a total proportion of 0.5714.

To obtain the results presented here, 100,000 or more independent replicates were performed. For each replicate the sharing among different kinds of relatives was computed, so for a founder full-sib family, the degree of sharing of, for example, uncle and nephew (or aunt and nephew, etc, because only autosomes were simulated), great-uncle and great-nephew, and cousins of degree up to third cousins were sampled successively. Although this induced sampling correlations, these were trivial because replicates were numerous and independent. Simulations were performed independently for chromosomes of different length and for three different founder relationships: linear descendants, full-sib–based, and half-sib–based (Table 1).

### Distribution of shared segments

#### Numbers of shared segments:

We provide examples to illustrate the kind of data available from the simulation for a map length of an “average” human chromosome of 1.632 M (based on Kong *et al.* 2004). Table 2 shows the distribution of numbers of shared segments (*n*_s_) for a range of relationships from a full-sib base and for a more limited number of half-sib–derived and lineal relationships. For this length of chromosome there is a less than 1% chance that uncle and nephew share no genome and approximately 15%, 35%, and 31% probability that they share 1, 2, and 3 regions, respectively. For half-sibs, who have the same Wright’s relationship (1/4) as uncle–nephew, the probabilities are 2%, 25%, 41%, and 24%, respectively. Of course, more distant relatives share fewer and smaller regions. For longer chromosomes (in terms of map length or expected number of recombinations) than shown in Table 2, the expected number of shared regions increases and length of individual segments decreases.

**Table 2:** Examples from 100,000 simulated replicates of the distribution of the numbers (*n*_s_) of genomic segments shared by relatives on a chromosome of 1.632 M

R	UN	GUGN	C	C1R	2C	2C1R	3C	HS	HUN	GPO	GGPO
*R*	1/4	1/8	1/8	1/16	1/32	1/64	1/128	1/4	1/8	1/4	1/8
*n*_s_	Number of replicates										
0	921	17,696	9976	33,823	55,541	71,901	83,006	1826	19,559	9965	28,052
1	14,948	29,825	29,729	33,471	27,620	19,682	12,902	24,700	34,913	54,387	43,923
2	35,379	28,580	32,644	20,836	11,902	6317	3180	41,257	28,856	30,210	22,223
3	31,041	16,286	19,168	8677	3789	1647	764	24,284	12,655	5046	5149
4	13,556	5940	6719	2526	934	362	122	6675	3379	386	610
5	3501	1408	1515	561	184	82	22	1150	579	6	39
6	587	237	231	95	28	8	3	101	57	0	4
7	65	26	17	11	2	1	1	6	2	0	0
8	2	2	1	0	0	0	0	1	0	0	0
9	0	0	0	0	0	0	0	0	0	0	0

UN, uncle–nephew; GUGN, great-uncle–great-nephew; C, cousin; C1R, cousin once removed; 2C, second cousin; 2C1R, second cousin once removed; 3C, third cousin; HS, half-sib; HUN, half-uncle–nephew; GPO, grandparent–offspring; GGPO, great-grandparent–great-grandoffspring.

#### Positions of shared segments:

Information also can be obtained from position of the shared regions, specifically whether they include the chromosome ends. Examples of the distribution of shared regions on the chromosome according to their position, specifically whether they include both, one, or no ends of the chromosome (*p*_s_ = 2, 1, 0, respectively), are shown in Table 3. A single region sharing both ends rarely occurs unless the relationship is close, and the proportion sharing at neither end increases as the relationship becomes more distant. Half-sibs are more likely to share regions including both chromosome ends than are uncle and nephew.

**Table 3:** Examples using simulations as in Table 2 of the distribution of numbers (*n*_s_), positions (*p*_s_), mean, and SD of total length of genomic regions shared by relatives on a chromosome of 1.632 M

			UN			C			2C			HS		
*n*_S_	*p*_S_		N	Mean	SD	N	Mean	SD	N	Mean	SD	N	Mean	SD
0			921			9976			55,541			1826		
1	2		947	1.000	0.000	43	1.000	0.000	0	—	*—*	1936	1.000	0.000
	1		7041	0.508	0.288	8888	0.211	0.196	6007	0.110	0.110	12,409	0.502	0.289
	0		6960	0.328	0.237	20,798	0.162	0.150	21,613	0.096	0.095	10,355	0.336	0.236
2	2		7044	0.675	0.234	1886	0.407	0.245	177	0.228	0.172	10,122	0.665	0.236
	1		18,878	0.500	0.226	14,514	0.307	0.190	3704	0.199	0.137	22,119	0.501	0.224
	0		9457	0.398	0.202	16,244	0.260	0.162	8021	0.177	0.120	9016	0.402	0.201
3	2		9768	0.605	0.201	2494	0.435	0.204	148	0.295	0.186	9152	0.600	0.200
	1		15,811	0.503	0.191	9837	0.366	0.170	1500	0.271	0.145	11,858	0.497	0.189
	0		5462	0.428	0.178	6837	0.317	0.155	2141	0.237	0.124	3274	0.428	0.176
4	2		5467	0.572	0.177	1411	0.457	0.182	83	0.405	0.174	3109	0.569	0.173
	1		6515	0.500	0.169	3486	0.402	0.161	418	0.336	0.148	2978	0.501	0.167
	0		1574	0.444	0.158	1822	0.361	0.147	433	0.294	0.126	588	0.440	0.159
>4	*All*		4155			1764			214			1258		

UN, uncle; C, cousin; 2C, second cousin; HS, half-sib.

*p*_s_=2, 1, 0 denotes sharing at both, one, and neither end of the chromosome, respectively.

#### Lengths of shared segments:

The expected proportion of genome shared (*i.e.*, 2*R*) is of course the same as the overall length of shared regions expressed as a proportion of the genome length, but the distribution of the lengths of the total and of individual shared segments depends on the pedigree **R**. Examples are also given in Table 3 for the mean and SD of the total length actually shared, expressed as a proportion of the chromosome length *l* = 1.632 M, as a function of whether the shared regions include zero, one, or two chromosome ends. A special case is when *n*_s_ = 1 and *p*_s_ = 2, when the length is invariant because the whole chromosome is shared.

### Summary of simulated statistics

Because the numbers (*n*_s_) and positions (*p*_s_) are discrete valued variables, to facilitate subsequent analysis total length shared on the chromosome also was summarized in discrete values, namely as the number of tenths of the chromosome shared (*t*_s_): if *x* (>0) is the length shared, then for *t*_s_ = 1: 0 < *x* ≤ 0.1, *t*_s_ *=* 2: 0.1 < *x* ≤ 0.2; …; *t*_s_ = 10: 0.9 < *x* ≤ 1.0. The distribution of the length of individual shared segments conditional on the numbers, positions, and total length shared on each chromosome was not included in subsequent analyses because it contains no additional information. For example, if there are two shared segments of total length *x*, then the relative lengths *y* and *x* – *y* tell us nothing about the numbers of generations apart. Although shown by simulation, on reflection it is obvious because the distribution is uniform.

To simulate the 22 human autosomes, map lengths were simplified into five classes, based on the data of Kong *et al.* (2004), and previously were used for illustration (Figure 5 of Hill and Weir, 2011), namely two chromosomes of 0.75 M, eight chromosomes of 1.25 M, six chromosomes of 1.75 M, four chromosomes of 2.1 M, and two chromosomes of 2.75 M, totaling 35.9 M. Simulation also was undertaken assuming 22 chromosomes each of 1.632 M, *i.e.*, with the same average length as in the model using five lengths. As shown later, there is little difference in predictions of discriminating ability between the five-length and one-length models, so further subdivision of chromosome lengths to more closely match those for humans for analysis would have little impact on calculations or conclusions. This does not, however, imply that individual lengths should be ignored in analyses of real data.

As inferred from Hill and Weir (2011), from variances of actual relationship and also from simulations, for half-sib–based relationships the distribution of shared regions (*n*_S_, *p*_S_, and *t*_S_) depends only on Wright’s numerator relationship *R*. For example, it is the same for half-cousins and half-great-uncle–great-nephew relationships (both *R* = 1/16), and for half second cousins and half-cousins twice removed (*R* = 1/64). Similarly, for full-sib–based relationships, the distribution is the same for second cousins and first cousins twice removed (*R* = 1/32), but is not the same for great-uncle–great-nephew and cousins (*R* = 1/8).

### Likelihood ratios

#### Computation:

Let *k* denote a specific realization {*n*_s_, *p*_s_, *t*_s_} of genome sharing on a specified chromosome, and let *P***_R_**(*k*) denote the probability of this outcome dependent on the chromosome length and conditional on the relationship being **R** (*e.g.* half-sibs). If, for example, only information on *n*_s_ is used, then the realization is simply {*n*_s_}. The contribution provided by the observation *k* to the log likelihood ratio *λ*(**A : B)** for relationships **A** and **B** is then log*P***_A_**(*k*) **−** log*P***_B_**(*k*) using the logarithm inter alia because it has better sampling properties. We use the simulation results to obtain these probabilities, computed simply as the proportion of replicates with the appropriate outcome. Thus, using only data on *n*_s_, for example, and assuming three shared segments, then *λ*(UN : HS) ∼ ln(0.310/0.243) = 0.245. and *λ*(UN : GUGN) ∼ ln(1.91) = 0.645 (Table 2). Because segregation over chromosomes is independent, the total log likelihood ratio is obtained by summing contributions to the log likelihood ratio from different chromosomes, using probabilities appropriate to the map length and realization for each chromosome. If there is previous information regarding the relationships from nongenetic data and these can be quantified, then Bayes theorem can be used straightforwardly to compute posterior probabilities of alternative relationships. Otherwise, application is context-dependent, and we discuss that subsequently.

#### Moments:

Although any testing is situation-specific, we can investigate the properties of the log likelihood ratio as a function of the data used and possible relationships to be compared. Thus, we consider its moments, specifically its mean and variance. If the real relationship is **A**, then the contribution to the mean from a single chromosome is as follows in equation 1:

$${\text{E}}_{A}\left[\lambda \left(A:B\right)\right]=\Sigma_{k}P_{A}\left(k\right)\left[\log P_{A}\left(k\right)-\log P_{B}\left(k\right)\right],$$

and there is an equivalent formula for the variance. The overall mean and variance of *λ* are obtained by summing contributions over chromosomes. We also compute its skew and kurtosis.

The mean *λ* is the (directed) Kullback-Leibler distance between the two distributions *P*_**A**_ and *P*_**B**_ (Kullback and Leibler 1951; Burnham and Anderson 2001). This “distance” is not symmetric, *i.e.*, in general, E**_A_**[*λ*(**A** : **B**)] ≠ E**_B_**[*λ*(**B** : **A**)]. Subsequently, we tabulate values over the correct distribution (*i.e.*, real relationship) such that they are positive.

Because the numbers of shared segments and their positions are count data and because lengths shared were analyzed similarly as discrete variables, the numbers in each defined class *k* have a multinomial distribution with parameters estimated from the simulation results. In computing the moments of *λ*, the expected probabilities *P***_R_**(*k*) were assumed to have been estimated by simulation with negligible error. If the estimate from simulation of *P***_A_**(*k*) was not zero but that of *P***_B_**(*k*) was zero, then in computing the term *P***_A_**(*k*)[log*P***_A_**(*k*) **–** log*P***_B_**(*k*)**],** it was assumed that *P***_B_**(*k*) = 1/(2*N*), where *N* was the number of replicates simulated. This term becomes important only when the distributions differ greatly [in which case E(*λ*) is already large] and when expected numbers in cells become very small. To reduce errors such as this due to simulation, because data regarding numbers of segments itself included data regarding lengths, results given utilizing *n*_s,_ *p*_s_, and *t*_s_ used all three for 1 ≤ *n*_s_ ≤ 4, but only *n*_s_ and *p*_s_ for *n*_s_ > 4.

The ability to discriminate between alternative relationships using the likelihood ratio depends on the distribution of *λ*, mainly on the relative sizes of its mean and SD, so we tabulate E(*λ*)/SD(*λ*). Because there is replication of observations across chromosomes, SDs were computed over the aggregate, and therefore might be regarded as standard errors, but we retain the SD notation. We also found that λ typically has close-to-normal form.

## Results

### Moments of log likelihood ratios

#### Expectation:

Information available for contrasting relationships expressed as expected log likelihood ratios [E(*λ*), Kullback-Leibler distances] are provided in the upper part of Table 4 for a subset of relationships using the full simulated data for numbers (*n*_s_), positions (*p*_s_), and lengths (*t*_s_) of shared segments. In these and subsequent tables, rows denote the real relationship and columns denote the hypothesized relationship. Values of E(*λ*) for all 19 relationships analyzed and incorporating successively more information are given in Appendix Table A1 (using *n*_s_ only), Table A2 (using *n*_s_ and *p*_s_), and Table A3 (using *n*_s_, *p_s_*, and *t*_s_, *i.e.*, as in Table 4). In all these Tables, values were computed from simulation runs for each of the designated five map lengths (0.75, 1.25. 1.75, 2.10, and 2.75 M), each replicated 100,000 times, *i.e.*, as weighted averages over a total of 500,000 replicates.

**Table 4:** Expected log likelihood ratio, E(*λ*) (upper), and its SD, SD(*λ*) (lower), for a subset of relationships using information on numbers (*n*_s_), positions (*p*_s_), and total lengths (*t*_s_) of shared segments

*R*	1/4			1/8			1/32			1/128		
R	UN	HS	GPO	C	HUN	GGPO	2C	HC1R	G4PO	3C	H2C1R	G6PO
	E(*λ*)											
UN	0.00	2.12	27.91	14.85	14.52	26.08	64.08	60.25	61.21	110.08	107.37	105.92
HS	1.92	0.00	13.79	16.24	11.98	16.23	64.27	58.20	55.02	107.60	103.87	101.06
GPO	20.50	11.08	0.00	32.67	19.68	9.94	72.61	63.35	53.23	105.94	101.19	97.15
C	14.50	13.00	26.49	0.00	2.52	12.58	19.46	20.21	24.32	51.41	50.67	51.94
HUN	18.91	13.58	14.86	2.52	0.00	3.63	17.39	15.71	16.27	45.22	43.35	42.63
GGPO	28.41	19.29	8.20	10.41	3.02	0.00	20.89	17.01	13.89	44.29	41.59	39.12
	SD(*λ*)											
UN	—	2.16	8.50	5.40	4.56	6.93	10.28	9.35	8.93	12.15	11.87	11.52
HS	1.86	—	5.81	6.19	4.51	5.11	11.47	10.27	9.14	13.26	12.88	12.50
GPO	5.34	4.16	—	9.42	7.20	4.77	15.55	14.18	12.35	17.51	16.92	16.78
C	5.17	4.43	7.21	—	2.24	5.51	6.18	5.97	6.71	10.43	10.02	9.99
HUN	6.71	5.40	4.93	2.23	—	2.99	7.04	6.23	6.04	11.56	10.96	10.58
GGPO	7.64	6.49	3.57	4.17	2.26	—	8.75	7.54	6.28	12.91	12.21	11.51

UN, uncle–nephew; HS, half-sib; GPO, grandparent–offspring; C, cousin; HUN, half-uncle–nephew; GGPO, great-grandparent–great-grandoffspring; 2C, second cousin; HC1R, half-cousin once removed; G4PO, great-great-great-grandparent–great-great-great-grandoffspring; 3C, third cousin; H2C1R, half second cousin once removed; G6PO, great-great-great-great-great-grandparent–great-great-great-great-great-grandoffspring.

Lengths utilized only up to *n*_s_ = 4 assuming a model human autosomal genome comprising 2 chromosomes of length 0.75 M, 8 chromosomes of length 1.25 M, 6 chromosomes of length 1.75 M, 4 chromosomes of length 2.1 M, and 2 chromosomes of length 2.75 M. Rows denote the real relationship (A), columns denote the hypothesized relationship (B), and elements are E_A_[*λ*(A : B)] (equation 1).

It was seen that E(*λ*) is small when relationships are distant and of similar magnitude (Table 4, upper part), *e.g.*, second cousins and half-cousins once removed (for both of which *R* = 1/32). Although Kullback-Leibler distances are not symmetric, the reciprocal cases here are usually close but not identical in value, so only half the pairs of assumed relationships are included in Table 4 (but all are in the Appendix Tables). E(*λ*) is typically higher when the likelihood ratio is conditional on the higher relationship of the two, presumably because there is a wider distribution of numbers and lengths of segments shared among close relatives and therefore there is more information in the data.

The increment in E(*λ*) by incorporating position and length can be substantial for comparisons involving quite closely related individuals (Appendix Table A1, Table A2, and Table A3). As they become distant, *e.g.*, half-cousins *vs.* third cousins, the absolute and proportional increase is small. First, few shared segments are at the ends of chromosomes and the coefficient of variation in length shared decreases as the number of segments shared increases.

### Expectation *vs.* sampling error

SDs of *λ* values using all information (*n*_s_, *p*_s_, *t*_s_) are given in Table 4 (lower) for a number of relationships. Examples of E(*λ*)/SD(*λ*) for two subsets of relationships, one including pairs of high relationships (1/16 ≤ *R* ≤ 1/4 in Table 5) and the other including pairs of more distant relationship (1/128 ≤ *R* ≤ 1/16 in Table 6). Later, we discuss the interpretation of these values and show that the ratio is, at least approximately, a noncentrality parameter determining the probability of misassignment. Approximately, a value of 2.0 or more indicates a pair of relationships that can be distinguished with reasonable confidence. Full data fitting different amounts of information are given for SD(*λ*) in Appendix Table A4, Table A5, Table A6 and for E(*λ*)/SD(*λ*) in Appendix Table A7, Table A8, and Table A9. It is seen that SD(*λ*) tends to increase along with E(*λ*) as relationships become more different, *e.g.*, uncle–nephew *vs.* half-sib and *vs.* cousin (Table 5), and therefore E(*λ*)/SD(*λ*) diverges less rapidly than E(*λ*).

**Table 5:** Ratio of expected log likelihood ratio to its SD, E(*λ*)/SD(*λ*), using information on numbers, positions, and lengths of shared segments, as in Table 4: Sets of closely to moderately related pairs

		1/4			1/8				1/16		
*R*	R	UN	HS	GPO	GUGN	C	HUN	G2PO	C1R	HC	G3PO
		E(*λ*)/SD(*λ*)									
1/4	UN	—	0.98	3.28	2.95	2.75	3.18	3.76	4.69	5.04	5.40
	HS	1.03	—	2.37	2.62	2.62	2.66	3.17	4.24	4.37	4.77
	GPO	3.84	2.66	—	3.13	3.47	2.73	2.09	3.96	3.64	3.39
1/8	GUGN	2.64	2.63	3.27	—	0.70	0.56	1.68	1.50	1.64	2.03
	C	2.80	2.94	3.67	0.73	—	1.13	2.28	1.86	2.22	2.69
	HUN	2.82	2.51	3.02	0.60	1.13	—	1.22	1.52	1.49	1.77
	G2PO	3.72	2.97	2.30	1.92	2.50	1.34	—	1.94	1.57	1.33
1/16	C1R	4.79	4.75	4.97	1.71	1.85	1.80	2.20	—	0.65	1.32
	HC	4.76	4.46	4.77	1.75	2.14	1.60	1.86	0.66	—	0.73
	G3PO	5.18	4.60	4.19	2.31	2.85	1.90	1.49	1.43	0.78	—

UN, uncle–nephew; HS, half-sib; GPO, grandparent–offspring; GUGN, great-uncle–great-nephew; C, cousin; HUN, half-uncle–nephew; G2PO, great-grandparent–great-grandoffspring; C1R, cousin once removed; HC, half-cousin; G3PO, great-great-grandparent–great-great-grandoffspring.

**Table 6:** Ratio of expected log likelihood ratio to its SD, E(*λ*)/SD(*λ*), using information on numbers, positions, and lengths of shared segments as in Table 5: Sets of more distantly related pairs

		1/16			1/32			1/64			1/128		
*R*	R	C1R	HC	G3PO	2C	HC1R	G4PO	2C1R	H2C	G5PO	3C	H2C1R	G6PO
		E(*λ*)/SD(*λ*)											
1/16	C1R	—	0.65	1.32	1.19	1.39	1.68	2.03	2.15	2.30	2.66	2.75	2.84
	HC	0.66	—	0.73	1.03	1.05	1.23	1.73	1.79	1.90	2.29	2.35	2.42
	G3PO	1.43	0.78	—	1.21	1.02	0.95	1.63	1.59	1.61	2.07	2.07	2.10
1/32	2C	1.28	1.23	1.46	—	0.42	0.83	0.86	0.99	1.17	1.46	1.55	1.64
	HC1R	1.47	1.16	1.24	0.43	—	0.46	0.74	0.78	0.91	1.28	1.33	1.40
	G4PO	1.90	1.37	1.07	0.88	0.48	—	0.81	0.72	0.72	1.18	1.19	1.22
1/64	2C1R	2.37	2.20	2.29	0.95	0.89	1.01	—	0.28	0.54	0.64	0.73	0.84
	H2C	2.48	2.17	2.11	1.09	0.88	0.87	0.29	—	0.30	0.55	0.59	0.67
	G5PO	2.72	2.26	2.01	1.35	1.02	0.82	0.57	0.31	—	0.55	0.52	0.54
1/128	3C	3.41	3.13	3.08	1.76	1.62	1.62	0.72	0.65	0.70	—	0.19	0.35
	H2C1R	3.49	3.12	2.96	1.85	1.63	1.54	0.82	0.67	0.63	0.19	—	0.20
	G6PO	3.65	3.18	2.89	2.00	1.71	1.52	0.98	0.77	0.62	0.37	0.21	—

C1R, cousin once removed; HC, half-cousin; G3PO, great-great-grandparent–great-great-grandoffspring; 2C, second cousin; HC1R, half-cousin once removed; G4PO, great-great-great-grandparent–great-great-great-grandoffspring; 2C1R, second cousin once removed; H2C, half second cousin; G5PO, great-great-great-great-grandparent–great-great-great-great-grandoffspring; 3C, third cousin; H2C1R, half second cousin once removed; G6PO, great-great-great-great-great-grandparent–great-great-great-great-grandoffspring.

### Contributions from segment position and length

**Figure 1:**
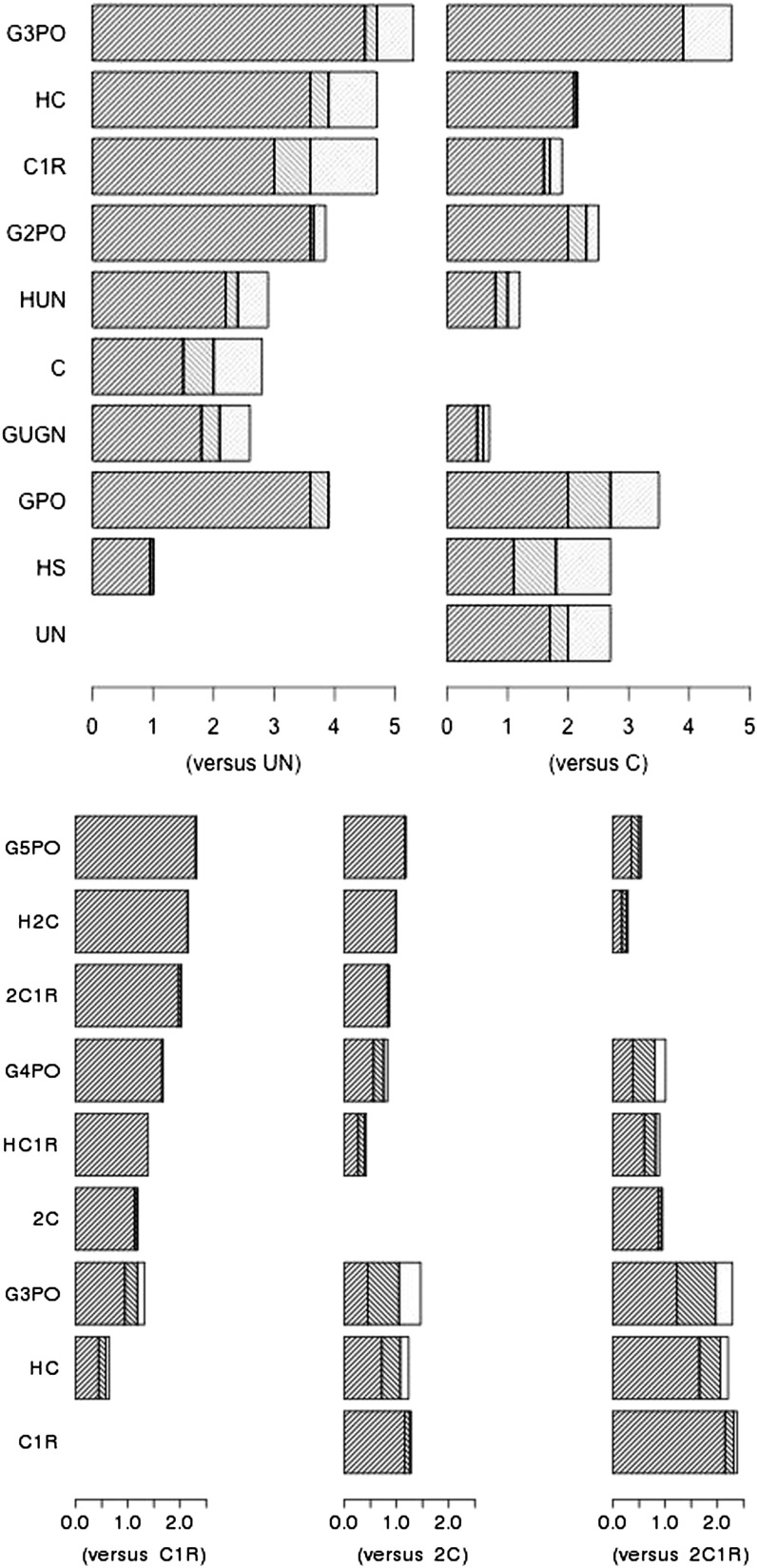
Cumulative contribution of sources of information to expected log likelihood ratio relative to its SD. E(*λ*)/SD(*λ*): number of segments only (*n*_s_ left), additional contribution from fitting position of segments (*p*_s_ center), and additional contribution from fitting length of segments (*t*_s_, right). (Upper) Sets of closely related pairs for real relationship uncle–nephew (UN) or cousin (C). (Lower) Sets of more distantly related pairs for cousins once removed (C1R), second cousins (2C), and second cousins once removed (2C1R).

The contributions of different components of the data to E(*λ*)/SD(*λ*) are illustrated in Figure 1 for some of the relationships in Table 5 and Table 6. It shows the ratio fitting only numbers of shared segments and shows the increments in the ratio by fitting positions and then lengths. A high proportion [in some cases almost all the information as judged by E(*λ*)/SD(*λ*)] is contained in the number of shared segments. A little more is added by including position, but only for close relationships when chromosomes ends are likely to be shared (Table 3). More information is obtained by incorporating length of chromosome shared, although not with a clearly defined pattern over relationships.

### Approximating likelihoods

#### Equal chromosome lengths:

To facilitate analysis of the distribution of log likelihood ratios, we consider a computational simplification, namely assuming all chromosomes have the same length rather than ranging over five different lengths. Hence, data also were simulated using a larger number of replicates (300,000) for chromosomes of length 1.632 M, the mean of those simulated previously, and likelihood ratios computed for genomes with 22 such chromosomes. Very similar values of E(*λ*), SD(*λ*), and, consequently, E(*λ*)/SD(*λ*) as those in Appendix Tables A1 through A9 were obtained. Results in Appendix Table A10 for E(*λ*)/SD(*λ*) enable comparison directly with those in Appendix Table A9 computed using the five chromosome lengths model. In summary, of the 342 off-diagonal comparisons of E(*λ*)/SD(*λ*) for the 19 relationships, only 32 deviated by more than 2% and of these 32, E(*λ*)/SD(*λ*) exceeded 1.0 in only 11, *i.e.*, large proportional differences typically occurred when absolute differences were small.

#### Replication:

Because differences in moments of *λ* ascribed to different models can arise from differences in expectation and from sampling in the simulation, a further run of 300,000 replicates for chromosomes of length 1.632 M as in Appendix Table A10 was undertaken (results not shown). The differences in E(*λ*)/SD(*λ*) between the replicates were very small; of the 342 off-diagonal comparisons, only 11 differed by more than 2%, and of those E(*λ*)/SD(*λ*) exceeded 1.0 in only 5. The main results (*e.g.*, Table 4, Table 5, Table 6 and corresponding Appendix Tables) computed for five lengths of chromosome involved a total of 500,000 unequally weighted runs, rather than 300,000 equally weighted runs (as we performed), so we conclude that sufficient replication was used.

### Higher moments and distributions of log likelihood ratios

**Figure 2:**
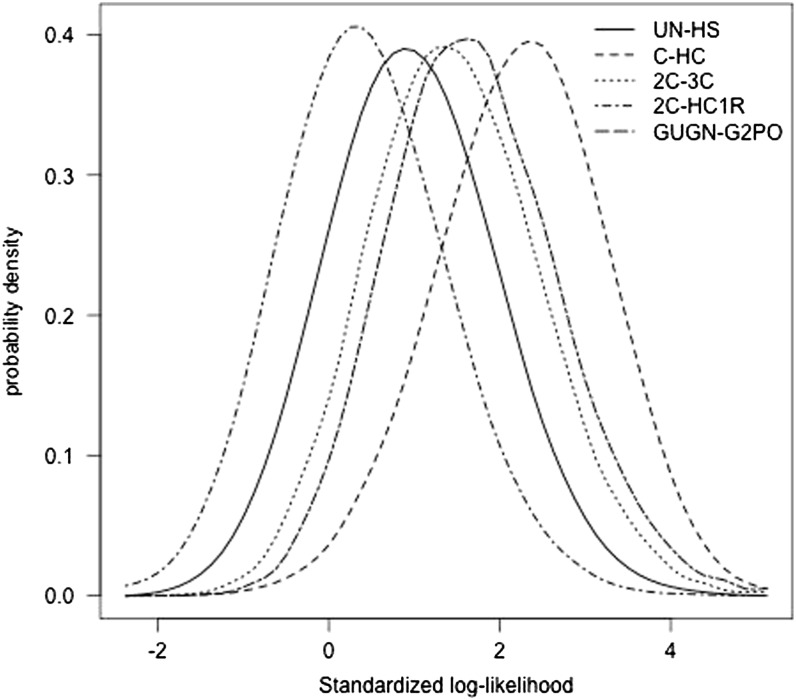
Distribution of log likelihood ratio, expressed relative to its SD, *λ*/SD(*λ*) using information on numbers, positions, and lengths of shared segments for examples of alternative pedigree relationships, *e.g.*, real relationship uncle–nephew hypothesized relationship half-sib (UN-HS). C, cousin; HC, half-cousin; 2C, second cousin; 3C, third cousin; HC1R, half-cousin once removed; GUGN, great-uncle–great-nephew; G2PO, great-grandparent–great-grandoffspring. Model of 22 chromosomes, each of 1.632 M.

To simplify calculations, and in view of these results showing a good approximation of likelihood statistics computed for a model of chromosomes of equal length as that for chromosomes of different lengths, higher moments and distribution of *λ* were computed assuming all chromosomes had length 1.632 M (from simulations as in Appendix Table A10). Coefficients of skew and kurtosis are given in Appendix Table A11 and Table A12, respectively, for a subset of relationships. In general, both coefficients are small, indicating closeness to a normal distribution. The kurtosis coefficient is generally smaller than the skew, and kurtosis tends to be seen only in the presence of skew. The largest skew generally is found when the true relationship is weak and the assumed relationship is stronger, in which case there is negative skew. Positive skew is found less often, but typically when the assumed relationship is weaker than the true relationship. The apparent near-normality is not unexpected because each sample is of size 22 and the central limit theorem applies (as it would to results simulated for samples from chromosomes of five different lengths). Examples of the distribution of the log likelihood ratio, scaled as *λ/*SD(*λ*), are given in Figure 2, showing near-“normal” form as anticipated in these particular examples.

### Approximations to sampling distributions

The results we have used have been based entirely on simulation. We investigate, however, theoretical results available that could be used to obtain some more simply computed but potentially less informative tests of pedigree relationship.

Based on work by Thomas *et al.* (1994), Huff *et al.* (2011) give an expression for the expected number of shared segments in the genome that, for a single chromosome, becomes the following equation (equation 2):

E(ns)=a(dl+1)(12)(d-1)

where *a* is the number of ancestors (1 for half-sib mating, 2 for full-sib mating), *d* is the total number of meioses separating ancestors and descendants (back to the grandparents), and *l* is the map length. For lineal descendents, numbers of shared segments are typically one-half those of half-sib descendents, and the expected number shared with the grandparent (or founder of a recurrent backcross line) is [(*d* − 1)*l* + 1](½)^(^*^d^*^−1)^, where *d* is the number of meioses back to the grandparent (*i.e.*, founder, hence terms in *d* − 1 because recombination to the parent is irrelevant). Thus, for example, *R* = 1/16 and *d* = 4 for full-sib–based (cousins once removed), half-sib–based (half cousins), and lineal descendents (great-great grandparent–great-great grandoffspring). The formulae do not apply to the cases of uncle–nephew, for which (surmised from simulations as in Table 2) E(*n*_s_) = (5*l* + 2)/4, or great-uncle–great-nephew, for which E(*n*_s_) = (7*l* + 2)/8. The mean numbers of shared segments from simulation agree (within sampling error) with prediction (Table 7).

**Table 7:** Mean and variance of number of shared segments on a chromosome of 1.632 M for different relationships obtained by simulation (300,000 replicates)*^a^*

*R*	FS-based	Mean	Variance	HS-based	Mean	Variance	Lineal	Mean	Variance
1/4	UN	2.539	1.170	HS	2.135	0.942	GPO	1.316	0.537
1/8	GUGN	1.678	1.482	HUN	1.475	1.187	GGPO	1.066	0.767
	C	1.885	1.311						
1/16	C1R	1.147	1.213	HC	0.941	1.020	G3PO	0.736	0.721
1/32	2C	0.674	0.845	HC1R	0.572	0.714	G4PO	0.471	0.543
1/64	2C1R	0.387	0.519	H2C	0.336	0.445	G5PO	0.287	0.358
1/128	3C	0.219	0.299	H2C1R	0.193	0.258	G6PO	0.169	0.217

FS, full-sib; HS, half-sib; UN, uncle–nephew; GPO, grandparent–offspring; GUGN, great-uncle–great-nephew; HUN, half-uncle–nephew; GGPO, great-grandparent–great-grandoffspring; C, cousin; C1R, cousin once removed; HC, half-cousin; G, great; G3PO, great-great-grandparent–great-great-grandoffspring; 2C, second cousin; HC1R, half-cousin once removed; G4PO, great-great-great-grandparent–great-great-great-grandoffspring; 2C1R, second cousin once removed; H2C, half second cousin; G5PO, great-great-great-great-grandparent–great-great-great-great-grandoffspring; 3C, third cousin; H2C1R, half second cousin once removed; G6PO, great-great-great-great-great-grandparent–great-great-great-great-grandoffspring.

a The mean number of shared segments from simulation agreed very closely with those expected from the formula. Of these 19 items, only three differences exceeded 0.001 and none exceeded 0.003.

Huff *et al.* (2011) also state that given *d*, the expected length of a shared segment is 1/*d*, based on the calculations of length surrounding a specific marker (Fisher 1949). They assume independence of numbers and length, implying from equation 2 that the expected total length of a chromosome shared is (*dl* + 1)(½)^(^*^d^*^−1)^/*d* for half-sib descendents. However, because the expected proportion of the genome shared is 2*R* = (½)^(^*^d^*^−1)^_,_ the mean total length shared is actually *l*(½)^(^*^d^*^−1)^. It is partitioned over the expected number (*dl* + 1)(½)^(^*^d^*^−1)^ of shared segments and, therefore, taking into account the finite length of the chromosome, the expected length of an individual segment is *l*/(*dl* + 1) = 1/(*d* + 1/*l*), not 1/*d*. These equations also hold for full-sib and lineal descendants. For example, for a chromosome of length 1.632 M, the expected lengths of a shared segment are 0.383 M, 0.277 M, and 0.217 M for half-sibs, half-uncle, and half-cousins, respectively, rather than 0.5 M, 0.333 M, and 0.25 M without the correction. The proportionate difference becomes smaller for more distant relatives, *e.g.*, 0.151 M rather than 0.167 M for half second cousins. For uncle–nephew and great-uncle–great-nephew, the expected lengths of individual segments on a chromosome are, from simulation, 2/(5*l* + 2) and 2/(7*l* + 2), respectively.

Huff *et al.* (2011) also made the simplifying assumption that the number of shared segments is Poisson-distributed, implying Var(*n*_s_) = E(*n*_s_) on individual chromosomes and the whole genome, but simulations show departures between mean and variance (Table 7). For a chromosome of length *l* = 1.632 M, the actual distribution is rather less dispersed than the Poisson for close relatives, but slightly more dispersed for more distant relatives. For cousins, for example, E(*n*_s_) = 1.882, V(*n*_s_) = 1.311, and the proportion sharing no segments is ∼10% (Table 2), but the Poisson expectation is ∼15%. Further, the distribution of shared segment lengths was assumed by Huff *et al.* to be independently exponentially distributed, in which case the coefficient of variation (CV) of the total length of shared segments on a chromosome would be proportional to 1/√*n*_s_. For close relations who may share a high proportion of the chromosome, the actual distribution is substantially underdispersed compared with the Poisson and the CV of total length shared deviates from the 1/√*n*_s_ prediction. As relationships get more distant, these predictions hold better.

### Using approximate sampling distributions to distinguish relationships

Because the predicted numbers (*n*_s_) of shared segments (Huff *et al.* 2011) have the correct mean, they provide a simple route to likelihood calculations without simulations. Further, as illustrated in Figure 1, most of the information can be obtained from the numbers of shared segments without using their positions and length. As the actual distribution departs from the Poisson (Table 7), however, there would be some reduction in discriminating power in computation of likelihoods, even from number of segments shared alone. To investigate this, we computed the log likelihood ratio for alternative types of relationships using data only regarding *n*_S_ assuming it is Poisson-distributed, and we computed its mean and SD using the actual frequency distribution obtained from simulation. For simplicity, we assumed 22 chromosomes each of length 1.632 M. Examples are given in Table 8 for the log likelihood ratio computed using both the Poisson and the actual distributions.

**Table 8:** Expected log likelihood ratio, E(*λ*), and ratio to its SD, E(*λ*)/SD(*λ*), using data from numbers of shared segments (*n*_s_) only computed from simulated data and also from the Poisson assumption, but with weights as for the simulated (actual) data

		Simulated		Poisson		Simulated		Poisson		Simulated		Poisson	
		E	E/SD	E	E/SD	E	E/SD	E	E/SD	E	E/SD	E	E/SD
*R*	1/4	UN				HS				GPO			
1/4	UN	0.00	—	0.00	—	1.82	0.90	0.81	0.91	26.54	2.43	9.81	2.94
1/8	GUGN	11.01	1.86	3.66	1.54	6.28	1.41	1.15	0.84	8.36	1.25	1.01	0.73
1/8	C	5.64	1.42	2.05	1.27	2.30	0.89	0.33	0.49	9.05	1.33	2.38	1.24
*R*	1/8	C				HUN				GGPO			
1/8	C	0.00	—	0.00	—	1.52	0.87	1.16	0.89	8.42	1.76	5.62	1.84
1/16	C1R	5.75	1.62	3.68	1.43	1.38	0.80	0.87	0.66	1.29	0.66	0.07	0.19
1/32	2C	16.22	2.93	11.35	2.56	7.73	2.02	6.00	1.78	3.90	1.39	1.83	0.92
*R*	1/32	2C				HC1R				G4PO			
1/32	2C	0.00	—	0.00	—	0.15	0.26	0.19	0.27	0.76	0.54	0.86	0.54
1/64	2C1R	1.35	0.88	1.59	0.85	0.66	0.60	0.75	0.56	0.29	0.38	0.17	0.26
1/128	3C	3.91	1.64	4.59	1.58	2.70	1.33	3.13	1.26	6.00	2.04	5.54	1.78

UN, uncle–nephew; HS, half-sib; GPO, grandparent–offspring; GUGN, great-uncle–great-nephew; C, cousin; C1R, cousin once removed; 2C, second cousin; 2C1R, second cousin once removed; 3C, third cousin.

Model of 22 chromosomes, each of 1.632 M. Actual relationships are in rows. Hypothesized relationships are in columns.

The log likelihood ratios remain zero when the real and assumed relationships are the same. In general, E(*λ*) is smaller when the Poisson approximation is used, but the proportional reduction is inconsistent. There are cases when it is larger, which seems illogical, but there is no guarantee *λ* decreases because the test is against a false hypothesis, with the actual distribution fitting closer to the Poisson with the wrong parameters. Because the SD is also substantially affected and typically is smaller, the ratio E(*λ*)/SD(*λ*) is often larger than that computed using the correct distribution obtained by simulation, but the pattern is not consistent. In view of this, such approximations should be used with care, and in any case we have provided an exact approach (strictly, more nearly exact, from replicate simulations).

### Extension to other species: impact of chromosome number and length

Results have been given for a model human genome of *c* = 22 autosomes with a total map length of *L* = 35.9 M; however, to assess how they need modifying for other species, we consider how *c* and *L* influence results. We have shown that a model of 22 chromosomes of equal average length (1.632 M) approximates that with lengths ranging from 0.75 M to 2.75 M, with most in mid range. Therefore, if chromosomes have similar mean length to those of humans and the distribution of lengths is no more dispersed, moments for different numbers of chromosomes can be predicted well by scaling as E(*λ*) ∝ *c* and E(*λ*)/SD(*λ*) ∝ √*c* because they are independent. To investigate the impact of wider variation in length we considered alternatives with total genome length 36 M, comprising 72 chromosomes each of 0.5 M or 12 chromosomes each of 3 M.

Ability to discriminate, expressed in terms of E(λ)/SD(*λ*), is given for some examples of relationship in Appendix Table A13 using either numbers of segments alone or all sources, *i.e.*, numbers, positions, and lengths. In summary, when there are many independent chromosomes, E(*λ*)/SD(*λ*) is generally higher than when there are few, particularly when Wright’s relationship *R* differs, because probabilities of ibd at individual loci are mostly uncorrelated with many small chromosomes. Independent loci do not provide evidence to distinguish relationships such as uncle–nephew and half-sibs having the same *R*. Information is contained in the distribution of number and length of shared segments, however, and the differences in E(*λ*)/SD(*λ*) between the 12 and 72 chromosome models for the same total map length are small, although generally higher for *c* = 72 when comparing relationships with different *R*. For relationships with the same *R* there is negligible difference, *e.g.*, real relationship half-sib, assumed uncle–nephew, E(*λ*)/SD(*λ*) = 0.97 for 72 chromosomes, 0.98 for 12 (Appendix Table A13) chromosomes, and 0.98 for 22 variable-length chromosomes (Table 5). Overall, therefore, the discriminating power clearly depends more on total amount of genome rather than on the individual chromosome lengths for the typical range of lengths in mammals.

## Discussion

### Inference

Although likelihood ratios are a natural way to describe the plausibility of alternative relationships, how to draw inferences from them is less clear-cut. Let **Ω** denote the set of all pedigree relationships **R** under consideration. Because this is a finite set of discrete elements, it removes some of the difficulties in assigning prior probabilities when, typically, these are neither specified nor easy to specify. Bayes theorem can then be used to combine likelihoods and prior probabilities to produce a posterior distribution over the elements of **Ω**. Unless some form of ordering, or measure of distance, is introduced in **Ω**, it is impossible to speak of means or variances of this distribution, but it will usually have a unique mode, and the corresponding relationship **R** will be our “best guess” at the true relationship. A confidence set could be obtained by ordering relationships by posterior probability and dropping relationships with the smallest probabilities until a desired probability level is achieved for the remainder.

Without prior probabilities, everything hangs on the likelihood. The likelihood function is defined on **Ω**, and the relationship **R** in **Ω** that produces the maximum value of the likelihood is the maximum likelihood estimate of the true relationship, corresponding to the posterior mode with a uniform prior. Without a distance measure, and with discrete relationship classes, standard asymptotic results for maximum likelihood estimates are not available. The distribution of the maximum likelihood estimate could be calculated by simulation, however, assuming any particular **R** to be true.

Any particular **R** can be tested as a null hypothesis against the general alternative that the true relationship is not **R** by using a maximum likelihood ratio test (McPeek and Sun, 2000). The set of those **R** in **Ω** for which this test is not significant at a given significance level constitutes a confidence set for the unknown relationship. McPeek and Sun (2000, p. 1079) point out that although the sampling distribution of log likelihood ratios for two fixed relationships is often close to a normal distribution (as we have shown previously; Figure 2, Appendix Tables A11 and A12), the sampling distribution of the maximized version tends to be skewed (the difference is between the estimate of **R** fixed or varying from sample to sample). Nevertheless, even in the normal case, simulation is required to obtain the mean and variance of the null distribution.

An issue that arises with both Bayesian and likelihood approaches is the completeness or otherwise of **Ω.** The true relationship might be one we neglected to consider; it might be bilinear, but not so detected (*e.g.*, paternal half-sibs and maternal second cousins), or an ancestor might be inbred so the probabilities of ibd sharing of descendents differ from those assumed here. Some relationships could be excluded based, for example, on ages of the individuals concerned, *e.g.*, some lineal or avuncular relationships.

If all that is required is to identify the “best guess” among all relationships under consideration, then we select the relationship with the largest likelihood, or the largest posterior probability. This can be regarded as a discrimination problem, with the relationships treated symmetrically. The two solutions correspond to the maximum likelihood or Bayes discriminate rules (Mardia *et al.* 1979), and the performance of such rules is judged by the set of misclassification probabilities.

Discriminating between two relationships amounts to choosing one if the log likelihood ratio *λ* > 0 and choosing the other if *λ* < 0. If the two relationships are **A** and **B**, and the distribution of *λ* is normal in each case, then the misclassification probabilities are Φ(−*m*_**B**_/*s*_**B**_) when we choose **A**, and Φ(−*m*_**A**_/*s*_**A**_) when we choose **B**, where *m*_**A**_ = E_**A**_[*λ*(**A** : **B**)], *i.e.*, the mean of *λ* when **A** is the true relationship, and *m*_**B**_ = E_**B**_[*λ*(**B** : **A**)] (= −E_**B**_[*λ*(**A** : **B**)]) when **B** is the true relationship and *s*_**A**_ and *s*_**B**_ are the corresponding SDs. Ratios of *m*/*s* for various pairs of relationship are in Table 5 and Table 6, with more in Appendix Tables A7–A9.

As an example, let us assume X dies intestate and a search locates one living relative, indisputably a half-cousin. Subsequently, Y appears claiming to be a cousin of X (but otherwise unrelated to Y), and thus is more closely related. Given only DNA data, can the claim be substantiated or disproved? There are two competing hypotheses: for **A**, Y is a cousin of X; and for **B**, Y is a half-cousin of X. To keep this argument simple, we discount other possible relationships. Given a prior probability that X and Y are cousins, the Bayesian approach provides a posterior probability, but it is not clear what a reasonable prior probability would be in the absence of any background information for Y. With the likelihood approach, we can clearly discriminate with confidence in this situation because both misclassification probabilities are small, ∼1.5% using Table 5, Φ(−2.22) ∼ 0.013, *i.e.*, if we decide half-cousins, and Φ(−2.14) ∼ 0.016 if we decide cousins.

Taking as a simple criterion a difference of 2 SD in log likelihood ratio as an indicator of discriminating ability (corresponding to a misclassification probability of approximately 0.02), it is seen that although it is possible to distinguish between a distant and a close relationship with high power, it is more difficult between relationships of the same degree (*R*), increasingly so as *R* becomes smaller (Table 5 and Table 6). There is little power to discriminate between relationships for which *R* is 1/64 or less; for example, the probability of correct assignment (based simply on sign of the log likelihood ratio) is approximately 3/4 for second cousins once removed *vs.* third cousins as E(*λ*)/SD(*λ*) ∼ 0.6. It is easier to distinguish lineal relationships, *e.g.*, great-great-great-grandparent–offspring from second cousins, than it is to distinguish second cousins from half-cousins once removed (for all of which *R* = 1/32) because the lineal recombination and transmission process differs more than that between half and full-sib descendants.

Without use of information as shown here regarding shared genomic regions and merely considering resemblance locus by locus, relationships such as uncle–nephew and half-sib cannot be distinguished at all. It is seen that E(*λ*)/SD(*λ*) ∼ 1, whichever relationship is the real one. Hence, the likelihood ratio will be in the correct direction approximately 5/6 of the time—not certainty at a level looked for in significance tests, but not valueless. For more distant pairs with the same *R*, the probability of correct assignment will decline; for second cousins and half-cousins once removed (*R* = 1/32), the probability declines to approximately 2/3. This illustrates the limitations of making decisions about the relationship between a pair of individuals even if based on full genomic data.

### Assumptions

Many assumptions have been made in this analysis. The first is that the number of shared segments is accurately recorded, and the main risk is that short segments are missed. In population studies, Browning and Browning (2013) and S.R. Browning (personal communication) report good power to detect segments of 1.5 cM and higher using dense SNP data and 1 cM or higher with sequence data. For exponentially distributed segment lengths of expected length *a* (cM), this would imply a probability of missing an individual segment of approximately 1.5/*a* (1/*a*) from SNP (sequence) data. For half-cousins, for example, the expected segment length is 21.7 cM for a chromosome of 1.632 M (see *Results* regarding approximations to sampling distributions), implying an approximately 7% chance of missing a random segment using SNPs, slightly less for closer relatives or using sequence data. Thus, there would be bias towards underestimating both Wright’s and pedigree relationship, but little in comparing relatives with the same *R*. For known relatives, however, as considered here, the probability would be expected to be much lower because the individuals are already identified as relatives and not trawled from the population. Errors in estimating segment length would be comparatively unimportant (Figure 1).

Errors therefore will not necessarily lead to wrong assignment but to miscalculation of the likelihood ratios. As Table 2 and Table 3 show, however, the pattern of numbers shared is unlikely to change greatly if the error rate is no more than a few percent, and the relative parameters for different relationships will remain approximately the same. A detailed analysis of consequences of errors is beyond the scope of this article, however.

Further assumptions made when information on chromosome length is included are that a Haldane mapping function is appropriate and that map length can be accurately inferred from physical length of the chromosome. We consider the number of segments and the probability that shared segments reach chromosome ends would depend little or not at all on the mapping function. Problems might be encountered in measuring the segment length distribution, converted to map units, before using the data and methods presented here. If there are major experimental technical problems in measuring lengths or concern about the mapping functions or conversion from physical length, then that information could just be ignored with, for most pairings of **R**, little impact on discriminating ability (Figure 1).

We also have taken no account of distant background relationship, assuming all genome sharing was due to recent common ancestry, whereas Huff *et al.* (2011) did so. Such sharing will bias predictions towards higher relationship. As in the example here, an extra rather than lost shared segment on one or two chromosomes will have little effect on likelihood calculations for fairly close relationships. Proportional errors become larger as relationships become more distant, but as results such as in Table 6 show, the power to discriminate among quite distant relationships is low in any case.

### General conclusions

The results presented here show what can, in theory, be achieved in determining pedigree relationships from information on genome sharing. No further information is, in principle, available from analysis at the individual locus level (except perhaps from sequencing and tracing point mutations in the pedigree). The low levels of expected likelihood ratios compared with their sampling error for pairs of quite distant relationships illustrate both how much variability in actual relationship in terms of shared genome comes from random Mendelian segregation and linkage and the consequent difficulty in assigning relationship.

## Supplementary Material

Supporting Information

## Appendix

Simulated data in supplementary files.

**Appendix Table A1:** Expectation of log likelihood ratio E(*λ*) for 19 relationships incorporating information only on numbers of shared segments

*R*		1/4			1/8				1/16			1/32			1/64			1/128		
	**R**	UN	HS	GPO	GUGN	C	HUN	G2PO	C1R	HC	G3PO	2C	HC1R	G4PO	2C1R	H2C	G5PO	3C	H2C1R	G6PO
1/4	UN	0.00	1.85	24.25	6.54	4.10	10.08	24.75	16.17	22.01	33.43	30.75	36.05	45.52	46.51	51.22	59.32	62.88	66.85	73.73
	HS	1.67	0.00	11.66	3.69	1.63	5.10	14.05	10.59	14.84	22.64	22.85	27.03	33.89	36.70	40.56	46.68	51.32	54.65	60.10
	GPO	17.22	9.10	0.00	4.81	5.08	3.03	2.30	5.04	6.23	7.84	11.08	13.13	15.56	19.87	22.13	24.81	30.17	32.28	34.94
1/8	GUGN	10.93	6.19	7.46	0.00	0.67	0.49	5.58	2.41	4.91	10.17	9.93	12.88	17.86	19.82	22.73	27.33	30.93	33.55	37.65
	C	5.73	2.36	8.29	0.58	0.00	1.48	8.30	4.92	8.21	14.62	14.51	18.01	23.85	26.13	29.47	34.76	38.80	41.74	46.43
	HUN	14.43	8.01	4.04	0.42	1.52	0.00	2.49	1.31	3.02	6.63	7.46	9.85	13.64	16.21	18.70	22.38	26.32	28.60	32.02
	G2PO	26.44	16.96	2.96	3.63	6.38	1.88	0.00	1.01	1.16	1.96	3.70	5.01	6.75	9.61	11.26	13.34	17.22	18.86	20.96
1/16	C1R	26.54	17.95	6.59	2.39	5.70	1.41	1.29	0.00	0.41	2.30	2.59	4.07	6.55	8.36	10.13	12.74	15.76	17.49	19.98
	HC	34.30	24.31	8.26	4.64	9.12	3.18	1.24	0.39	0.00	0.70	1.01	1.93	3.53	5.14	6.47	8.37	11.05	12.43	14.34
	G3PO	43.02	31.56	10.59	7.92	13.53	5.94	2.05	1.77	0.57	0.00	0.42	0.77	1.42	2.89	3.78	4.93	7.34	8.37	9.65
1/32	2C	48.36	36.69	14.75	9.47	16.05	7.70	3.87	2.47	0.99	0.43	0.00	0.15	0.77	1.50	2.23	3.30	5.02	5.91	7.11
	HC1R	53.86	41.54	17.34	11.71	19.02	9.78	5.19	3.72	1.82	0.75	0.14	0.00	0.23	0.71	1.21	1.96	3.38	4.08	5.02
	G4PO	59.59	46.58	20.01	14.27	22.29	12.16	6.72	5.29	2.98	1.35	0.63	0.20	0.00	0.29	0.55	0.97	2.13	2.65	3.30
1/64	2C1R	66.19	52.77	24.64	17.10	26.04	15.05	9.32	7.17	4.51	2.67	1.35	0.66	0.28	0.00	0.06	0.30	0.89	1.25	1.73
	H2C	69.70	55.98	26.75	18.78	28.15	16.68	10.63	8.33	5.46	3.39	1.91	1.08	0.52	0.06	0.00	0.09	0.48	0.74	1.10
	G5PO	73.30	59.25	28.87	20.59	30.36	18.42	12.00	9.61	6.53	4.22	2.60	1.62	0.87	0.25	0.08	0.00	0.22	0.38	0.60
1/128	3C	79.19	64.79	33.13	23.54	34.03	21.40	14.72	11.82	8.45	5.95	3.91	2.72	1.80	0.77	0.43	0.20	0.00	0.03	0.12
	H2C1R	81.26	66.73	34.54	24.64	35.36	22.49	15.67	12.66	9.19	6.59	4.44	3.18	2.18	1.04	0.64	0.35	0.03	0.00	0.03
	G6PO	83.33	68.64	35.91	25.77	36.71	23.61	16.62	13.53	9.95	7.25	5.01	3.68	2.59	1.36	0.90	0.53	0.10	0.03	0.00

UN, uncle–nephew; HS, half-sib; GPO, grandparent–grandoffspring; GUGN, great-uncle–great-nephew; C, cousin; HUN, half-uncle–nephew; G2PO, great-grandparent–grandoffspring; C1R, cousin once removed; HC, half-cousin; G3PO, great-great-grandparent–grandoffspring; 2C, second cousin; HC1R, half-cousin once removed; G4PO, great-great-great-grandparent–grandoffspring; 2C1R, second cousin once removed; H2C, half second cousin; G5PO, great-great-great-great-grandparent–grandoffspring; 3C, third cousin; H2C1R, half second cousin once removed; G6PO, great-great-great-great-great-grandparent–grandoffspring.

**Appendix Table A2:** E(*λ*) as Table A1, but incorporating information on numbers and positions of shared segments

*R*		1/4			1/8				1/16			1/32			1/64			1/128		
	**R**	UN	HS	GPO	GUGN	C	HUN	G2PO	C1R	HC	G3PO	2C	HC1R	G4PO	2C1R	H2C	G5PO	3C	H2C1R	G6PO
1/4	UN	0.00	2.09	28.01	8.94	7.77	11.43	24.81	23.83	26.90	35.56	42.70	45.16	51.48	62.79	65.08	69.54	83.90	85.53	88.10
	HS	1.90	0.00	13.82	8.01	7.73	7.99	14.54	21.51	22.42	26.69	38.75	39.61	42.89	57.16	58.34	60.88	76.69	77.09	79.03
	GPO	20.47	11.07	0.00	18.27	22.39	14.06	7.50	29.95	25.68	21.28	42.97	39.89	37.61	56.14	55.19	54.36	70.68	68.71	70.07
1/8	GUGN	12.60	9.06	16.48	0.00	0.81	0.59	6.94	3.37	5.19	10.16	12.17	14.18	18.33	23.54	25.51	28.96	36.31	38.02	40.69
	C	8.53	6.79	20.40	0.72	0.00	1.94	10.78	5.38	8.27	14.80	16.00	18.76	24.01	28.94	31.46	35.76	43.16	45.26	48.61
	HUN	15.48	9.98	11.32	0.52	1.95	0.00	3.21	2.90	3.69	6.69	10.55	11.85	14.61	20.93	22.38	24.84	32.79	34.02	36.09
	G2PO	26.61	17.38	6.32	4.78	8.50	2.53	0.00	5.06	3.67	3.03	9.79	9.55	9.81	17.52	17.90	18.60	26.93	27.23	28.25
1/16	C1R	29.98	22.79	17.85	3.03	5.95	2.48	4.36	0.00	0.55	3.02	2.75	4.09	6.62	8.89	10.40	12.79	16.78	18.21	20.33
	HC	36.35	27.38	16.31	4.80	9.15	3.57	2.94	0.52	0.00	0.91	1.53	2.13	3.54	6.17	7.13	8.68	12.68	13.68	15.14
	G3PO	43.99	33.17	15.80	7.92	13.66	5.97	2.70	2.34	0.75	0.00	1.59	1.45	1.70	4.71	5.12	5.80	9.83	10.39	11.21
1/32	2C	51.29	40.62	23.22	10.27	16.46	8.84	6.51	2.56	1.32	1.30	0.00	0.20	1.00	1.56	2.23	3.32	5.20	6.01	7.13
	HC1R	55.90	44.36	23.88	12.11	19.18	10.42	6.94	3.72	1.93	1.20	0.19	0.00	0.29	0.90	1.28	1.97	3.75	4.33	5.13
	G4PO	60.88	48.45	24.81	14.42	22.31	12.43	7.75	5.33	2.99	1.51	0.81	0.25	0.00	0.68	0.78	1.06	2.75	3.11	3.59
1/64	2C1R	68.31	55.54	30.36	17.80	26.43	15.97	11.24	7.31	4.85	3.41	1.38	0.78	0.56	0.00	0.08	0.37	0.91	1.26	1.74
	H2C	71.32	58.12	31.39	19.23	28.37	17.30	12.04	8.39	5.65	3.88	1.92	1.12	0.66	0.08	0.00	0.11	0.55	0.77	1.10
	G5PO	74.46	60.83	32.51	20.84	30.46	18.79	12.98	9.62	6.61	4.49	2.62	1.63	0.92	0.32	0.10	0.00	0.36	0.46	0.64
1/128	3C	80.59	66.59	36.73	24.05	34.33	22.05	15.98	11.96	8.73	6.49	3.96	2.84	2.04	0.78	0.47	0.30	0.00	0.03	0.14
	H2C1R	82.40	68.20	37.59	25.01	35.57	22.98	16.67	12.74	9.38	6.99	4.46	3.25	2.34	1.05	0.66	0.40	0.03	0.00	0.04
	G6PO	84.21	69.81	38.41	26.03	36.84	23.95	17.38	13.57	10.07	7.52	5.02	3.71	2.68	1.36	0.90	0.55	0.13	0.04	0.00

UN, uncle–nephew; HS, half-sib; GPO, grandparent–grandoffspring; GUGN, great-uncle–great-nephew; C, cousin; HUN, half-uncle–nephew; G2PO, great-grandparent–grandoffspring; C1R, cousin once removed; HC, half-cousin; G3PO, great-great-grandparent–grandoffspring; 2C, second cousin; HC1R, half-cousin once removed; G4PO, great-great-great-grandparent–grandoffspring; 2C1R, second cousin once removed; H2C, half second cousin; G5PO, great-great-great-great-grandparent–grandoffspring; 3C, third cousin; H2C1R, half second cousin once removed; G6PO, great-great-great-great-great-grandparent–grandoffspring.

**Appendix Table A3:** E(*λ*) as Tables A1 and A2, but incorporating information on numbers, positions, and lengths of shared segments

*R*		1/4			1/8				1/16			1/32			1/64			1/128		
	**R**	UN	HS	GPO	GUGN	C	HUN	G2PO	C1R	HC	G3PO	2C	HC1R	G4PO	2C1R	H2C	G5PO	3C	H2C1R	G6PO
1/4	UN	0.00	2.12	27.91	13.09	14.85	14.52	26.08	38.27	35.57	40.31	64.08	60.25	61.21	88.69	85.90	84.30	110.08	107.37	105.92
	HS	1.92	0.00	13.79	13.41	16.24	11.98	16.23	38.95	33.30	32.76	64.27	58.20	55.02	87.77	83.56	79.11	107.60	103.87	101.06
	GPO	20.50	11.08	0.00	25.86	32.67	19.68	9.94	51.04	40.07	29.61	72.61	63.35	53.23	90.87	85.10	77.28	105.94	101.19	97.15
1/8	GUGN	16.77	13.43	20.74	0.00	1.06	0.70	7.78	5.57	5.80	10.19	17.20	16.71	19.23	31.52	30.67	31.64	46.24	45.04	45.34
	C	14.50	13.00	26.49	0.98	0.00	2.52	12.58	6.54	8.45	14.96	19.46	20.21	24.32	35.14	35.08	37.36	51.41	50.67	51.94
	HUN	18.91	13.58	14.86	0.64	2.52	0.00	3.63	6.24	4.93	6.86	17.39	15.71	16.27	31.18	29.41	28.90	45.22	43.35	42.63
	G2PO	28.41	19.29	8.20	5.84	10.41	3.02	0.00	11.52	7.10	4.27	20.89	17.01	13.89	32.58	29.39	26.25	44.29	41.59	39.12
1/16	C1R	38.53	31.72	26.69	4.40	6.75	4.53	8.14	0.00	0.88	4.27	3.15	4.12	6.80	10.20	10.87	12.84	19.08	19.37	20.76
	HC	42.21	33.50	22.38	5.20	9.30	4.36	4.92	0.84	0.00	1.23	2.91	2.58	3.58	8.98	8.61	9.21	16.77	16.30	16.58
	G3PO	47.74	37.11	19.70	7.95	13.73	6.10	3.46	3.57	1.09	0.00	4.57	3.00	2.20	9.59	8.30	7.48	16.25	15.12	14.33
1/32	2C	59.06	48.73	31.28	12.11	17.84	11.30	10.44	2.80	2.12	3.05	0.00	0.37	1.63	1.72	2.25	3.45	5.69	6.15	7.14
	HC1R	61.66	50.38	29.86	13.05	19.82	11.81	9.47	3.75	2.20	2.08	0.35	0.00	0.46	1.50	1.46	1.99	4.88	4.89	5.32
	G4PO	64.98	52.74	29.07	14.75	22.50	13.05	9.18	5.43	3.02	1.80	1.41	0.43	0.00	2.00	1.44	1.26	4.79	4.41	4.26
1/64	2C1R	74.15	61.64	36.42	19.48	27.81	18.12	14.42	7.74	5.77	5.04	1.47	1.12	1.33	0.00	0.17	0.67	0.97	1.26	1.81
	H2C	75.88	62.89	36.13	20.29	29.20	18.72	14.31	8.55	6.13	4.89	1.93	1.23	1.04	0.16	0.00	0.20	0.79	0.84	1.12
	G5PO	77.92	64.45	36.11	21.41	30.88	19.64	14.49	9.66	6.79	5.03	2.69	1.65	1.05	0.60	0.18	0.00	0.90	0.74	0.72
1/128	3C	84.50	70.68	40.80	25.31	35.42	23.62	18.21	12.39	9.50	7.73	4.12	3.21	2.71	0.83	0.62	0.62	0.00	0.07	0.28
	H2C1R	85.60	71.55	40.92	25.92	36.32	24.14	18.40	12.98	9.88	7.87	4.53	3.44	2.76	1.06	0.71	0.56	0.07	0.00	0.09
	G6PO	86.77	72.48	41.07	26.61	37.31	24.74	18.64	13.67	10.34	8.08	5.03	3.78	2.90	1.41	0.92	0.60	0.25	0.08	0.00

UN, uncle–nephew; HS, half-sib; GPO, grandparent–grandoffspring; GUGN, great-uncle–great-nephew; C, cousin; HUN, half-uncle–nephew; G2PO, great-grandparent–grandoffspring; C1R, cousin once removed; HC, half-cousin; G3PO, great-great-grandparent–grandoffspring; 2C, second cousin; HC1R, half-cousin once removed; G4PO, great-great-great-grandparent–grandoffspring; 2C1R, second cousin once removed; H2C, half second cousin; G5PO, great-great-great-great-grandparent–grandoffspring; 3C, third cousin; H2C1R, half second cousin once removed; G6PO, great-great-great-great-great-grandparent–grandoffspring.

**Appendix Table A4:** SD(*λ*) as Table A1 using information only on numbers of shared segments

*R*		1/4			1/8				1/16			1/32			1/64			1/128		
	**R**	UN	HS	GPO	GUGN	C	HUN	G2PO	C1R	HC	G3PO	2C	HC1R	G4PO	2C1R	H2C	G5PO	3C	H2C1R	G6PO
1/4	UN	0.00	2.02	8.10	2.75	2.43	3.77	6.93	4.23	5.05	6.98	5.55	6.13	7.50	6.64	7.08	8.11	7.65	7.97	8.73
	HS	1.74	0.00	5.43	2.02	1.47	2.49	4.86	3.29	3.93	5.39	4.65	5.11	6.15	5.78	6.14	6.94	6.77	7.04	7.69
	GPO	4.87	3.74	0.00	2.53	2.51	2.13	1.85	2.67	2.89	3.16	3.68	3.95	4.30	4.77	5.02	5.36	5.83	6.03	6.36
1/8	GUGN	5.87	4.40	4.87	0.00	1.24	1.07	4.10	2.18	3.18	5.02	4.37	5.06	6.35	6.20	6.73	7.71	7.85	8.26	9.03
	C	3.98	2.58	5.19	0.99	0.00	1.70	4.64	2.85	3.73	5.47	4.77	5.39	6.61	6.37	6.85	7.78	7.81	8.17	8.91
	HUN	6.34	4.87	3.29	0.85	1.76	0.00	2.56	1.55	2.36	3.78	3.64	4.23	5.24	5.42	5.88	6.67	7.01	7.37	8.02
	G2PO	7.51	6.32	2.69	2.15	3.12	1.68	0.00	1.28	1.47	1.91	2.59	3.00	3.52	4.17	4.53	5.01	5.68	5.97	6.40
1/16	C1R	8.68	7.25	4.03	2.16	3.55	1.73	1.84	0.00	0.93	2.44	2.30	2.93	3.96	4.23	4.73	5.52	5.99	6.37	7.02
	HC	9.45	8.15	4.45	2.91	4.34	2.55	1.62	0.86	0.00	1.31	1.43	2.00	2.86	3.32	3.78	4.45	5.05	5.41	5.98
	G3PO	9.63	8.55	5.01	3.44	4.82	3.20	2.05	1.65	0.96	0.00	0.90	1.24	1.71	2.45	2.83	3.29	4.05	4.36	4.77
1/32	2C	10.48	9.33	5.73	4.06	5.51	3.82	2.78	2.14	1.38	0.95	0.00	0.56	1.37	1.81	2.25	2.86	3.48	3.83	4.33
	HC1R	10.56	9.51	6.08	4.35	5.78	4.17	3.16	2.55	1.83	1.21	0.52	0.00	0.73	1.23	1.63	2.16	2.83	3.15	3.59
	G4PO	10.32	9.42	6.34	4.48	5.84	4.37	3.50	2.85	2.21	1.59	1.01	0.58	0.00	0.77	1.08	1.46	2.20	2.49	2.83
1/64	2C1R	10.50	9.63	6.66	4.90	6.21	4.79	3.97	3.34	2.72	2.17	1.54	1.10	0.73	0.00	0.36	0.83	1.43	1.71	2.08
	H2C	10.30	9.50	6.73	4.96	6.21	4.88	4.13	3.49	2.91	2.40	1.78	1.37	0.98	0.33	0.00	0.44	1.03	1.30	1.63
	G5PO	9.93	9.23	6.74	4.92	6.10	4.87	4.23	3.57	3.04	2.59	1.99	1.62	1.24	0.66	0.37	0.00	0.69	0.91	1.17
1/128	3C	9.59	8.95	6.68	5.03	6.11	4.99	4.41	3.81	3.33	2.93	2.38	2.04	1.71	1.15	0.88	0.61	0.00	0.24	0.52
	H2C1R	9.33	8.74	6.61	4.98	6.02	4.96	4.43	3.84	3.38	3.01	2.47	2.15	1.85	1.30	1.05	0.79	0.22	0.00	0.27
	G6PO	8.96	8.43	6.49	4.87	5.85	4.86	4.40	3.80	3.39	3.06	2.53	2.23	1.96	1.44	1.20	0.95	0.43	0.24	0.00

UN, uncle–nephew; HS, half-sib; GPO, grandparent–grandoffspring; GUGN, great-uncle–great-nephew; C, cousin; HUN, half-uncle–nephew; G2PO, great-grandparent–grandoffspring; C1R, cousin once removed; HC, half-cousin; G3PO, great-great-grandparent–grandoffspring; 2C, second cousin; HC1R, half-cousin once removed; G4PO, great-great-great-grandparent–grandoffspring; 2C1R, second cousin once removed; H2C, half second cousin; G5PO, great-great-great-great-grandparent–grandoffspring; 3C, third cousin; H2C1R, half second cousin once removed; G6PO, great-great-great-great-great-grandparent–grandoffspring.

**Appendix Table A5:** SD(*λ*) as Table A2 using information on numbers and position of shared segments

*R*		1/4			1/8				1/16			1/32			1/64			1/128		
	**R**	UN	HS	GPO	GUGN	C	HUN	G2PO	C1R	HC	G3PO	2C	HC1R	G4PO	2C1R	H2C	G5PO	3C	H2C1R	G6PO
1/4	UN	0.00	2.15	8.56	3.62	3.81	4.12	6.91	6.20	6.14	7.26	8.16	7.98	8.43	9.86	9.74	9.76	11.58	11.49	11.08
	HS	1.85	0.00	5.82	3.91	4.29	3.63	4.94	6.63	6.08	6.25	8.70	8.18	8.06	10.38	10.07	9.85	12.10	11.69	11.41
	GPO	5.35	4.17	0.00	7.16	8.12	6.28	4.21	10.71	9.28	7.62	13.08	11.81	10.84	14.31	13.81	13.30	15.64	14.71	15.07
1/8	GUGN	5.94	4.64	6.24	0.00	1.34	1.17	4.51	2.72	3.30	5.01	5.20	5.51	6.48	7.39	7.60	8.16	9.45	9.60	9.84
	C	4.37	3.50	6.95	1.13	0.00	1.97	5.23	3.07	3.76	5.50	5.30	5.65	6.66	7.26	7.47	8.07	9.09	9.24	9.50
	HUN	6.39	5.00	4.65	0.94	1.98	0.00	2.86	2.61	2.74	3.81	4.95	5.03	5.58	7.06	7.14	7.46	9.08	9.09	9.24
	G2PO	7.53	6.34	3.26	2.60	3.68	2.01	0.00	3.71	3.10	2.61	5.57	5.16	4.95	7.35	7.19	7.09	9.18	8.90	9.01
1/16	C1R	8.42	6.98	5.08	2.31	3.56	2.06	2.98	0.00	1.09	2.80	2.41	2.95	3.98	4.49	4.86	5.54	6.42	6.69	7.16
	HC	9.24	7.86	4.68	2.93	4.34	2.60	2.23	0.98	0.00	1.49	1.88	2.15	2.87	3.89	4.13	4.60	5.81	5.99	6.32
	G3PO	9.51	8.37	4.80	3.44	4.83	3.20	2.18	1.91	1.11	0.00	2.03	1.88	1.95	3.61	3.65	3.80	5.33	5.37	5.54
1/32	2C	9.93	8.63	5.05	4.00	5.45	3.75	2.97	2.15	1.50	1.50	0.00	0.66	1.58	1.87	2.26	2.87	3.60	3.90	4.34
	HC1R	10.13	8.94	5.26	4.31	5.74	4.10	3.16	2.55	1.85	1.41	0.59	0.00	0.82	1.44	1.70	2.17	3.10	3.32	3.67
	G4PO	10.01	9.00	5.53	4.46	5.83	4.33	3.42	2.86	2.21	1.62	1.14	0.65	0.00	1.29	1.35	1.55	2.70	2.83	3.04
1/64	2C1R	9.85	8.82	5.38	4.74	6.09	4.59	3.68	3.32	2.69	2.20	1.54	1.15	0.96	0.00	0.42	0.94	1.45	1.73	2.09
	H2C	9.77	8.82	5.54	4.84	6.14	4.72	3.85	3.48	2.89	2.38	1.78	1.38	1.06	0.38	0.00	0.49	1.13	1.33	1.63
	G5PO	9.52	8.69	5.67	4.85	6.07	4.77	4.00	3.57	3.03	2.56	1.99	1.62	1.25	0.73	0.41	0.00	0.92	1.04	1.21
1/128	3C	8.97	8.18	5.34	4.84	5.98	4.75	4.00	3.77	3.25	2.81	2.37	2.02	1.72	1.15	0.90	0.70	0.00	0.27	0.58
	H2C1R	8.80	8.07	5.38	4.83	5.92	4.76	4.07	3.80	3.32	2.91	2.47	2.14	1.84	1.30	1.05	0.82	0.25	0.00	0.31
	G6PO	8.53	7.87	5.40	4.76	5.79	4.72	4.10	3.79	3.35	2.98	2.53	2.23	1.95	1.44	1.20	0.96	0.47	0.26	0.00

UN, uncle–nephew; HS, half-sib; GPO, grandparent–grandoffspring; GUGN, great-uncle–great-nephew; C, cousin; HUN, half-uncle–nephew; G2PO, great-grandparent–grandoffspring; C1R, cousin once removed; HC, half-cousin; G3PO, great-great-grandparent–grandoffspring; 2C, second cousin; HC1R, half-cousin once removed; G4PO, great-great-great-grandparent–grandoffspring; 2C1R, second cousin once removed; H2C, half second cousin; G5PO, great-great-great-great-grandparent–grandoffspring; 3C, third cousin; H2C1R, half second cousin once removed; G6PO, great-great-great-great-great-grandparent–grandoffspring.

**Appendix Table A6:** SD(*λ*) as Table A3 using information on numbers, position, and length of shared segments

*R*		1/4			1/8				1/16			1/32			1/64			1/128		
	**R**	UN	HS	GPO	GUGN	C	HUN	G2PO	C1R	HC	G3PO	2C	HC1R	G4PO	2C1R	H2C	G5PO	3C	H2C1R	G6PO
1/4	UN	0.00	2.16	8.50	4.44	5.40	4.56	6.93	8.16	7.06	7.46	10.28	9.35	8.93	11.50	11.14	10.50	12.15	11.87	11.52
	HS	1.86	0.00	5.81	5.11	6.19	4.51	5.11	9.18	7.62	6.87	11.47	10.27	9.14	12.69	12.22	11.25	13.26	12.88	12.50
	GPO	5.34	4.16	0.00	8.26	9.42	7.20	4.77	12.88	11.00	8.74	15.55	14.18	12.35	16.77	16.22	15.22	17.51	16.92	16.78
1/8	GUGN	6.36	5.11	6.34	0.00	1.51	1.26	4.63	3.72	3.55	5.03	6.69	6.23	6.70	9.25	8.81	8.75	11.32	10.87	10.71
	C	5.17	4.43	7.21	1.34	0.00	2.24	5.51	3.51	3.81	5.55	6.18	5.97	6.71	8.52	8.17	8.33	10.43	10.02	9.99
	HUN	6.71	5.40	4.93	1.07	2.23	0.00	2.99	4.10	3.31	3.87	7.04	6.23	6.04	9.56	8.91	8.43	11.56	10.96	10.58
	G2PO	7.64	6.49	3.57	3.04	4.17	2.26	0.00	5.94	4.52	3.22	8.75	7.54	6.28	11.08	10.24	9.17	12.91	12.21	11.51
1/16	C1R	8.04	6.67	5.37	2.57	3.64	2.51	3.69	0.00	1.37	3.24	2.65	2.97	4.06	5.03	5.05	5.58	7.18	7.04	7.30
	HC	8.86	7.50	4.69	2.98	4.35	2.72	2.64	1.26	0.00	1.70	2.82	2.46	2.90	5.18	4.81	4.84	7.32	6.94	6.84
	G3PO	9.21	8.07	4.71	3.43	4.82	3.21	2.33	2.49	1.39	0.00	3.78	2.95	2.32	5.88	5.22	4.64	7.86	7.29	6.81
1/32	2C	8.66	7.36	4.48	3.90	5.32	3.64	3.13	2.19	1.72	2.08	0.00	0.88	1.96	2.01	2.28	2.95	3.89	3.98	4.36
	HC1R	9.07	7.87	4.55	4.23	5.67	3.99	3.11	2.55	1.90	1.68	0.82	0.00	1.02	2.02	1.88	2.20	3.82	3.67	3.79
	G4PO	9.19	8.16	4.87	4.42	5.81	4.27	3.33	2.86	2.20	1.69	1.60	0.89	0.00	2.48	2.02	1.76	4.06	3.72	3.50
1/64	2C1R	8.20	7.12	4.11	4.39	5.77	4.15	3.21	3.26	2.62	2.20	1.56	1.26	1.32	0.00	0.59	1.24	1.52	1.74	2.16
	H2C	8.37	7.38	4.34	4.59	5.93	4.39	3.41	3.45	2.83	2.32	1.78	1.40	1.20	0.56	0.00	0.65	1.45	1.42	1.66
	G5PO	8.39	7.52	4.64	4.70	5.95	4.56	3.66	3.56	3.00	2.50	2.00	1.61	1.28	1.05	0.58	0.00	1.64	1.42	1.33
1/128	3C	7.31	6.47	3.85	4.37	5.56	4.18	3.28	3.63	3.03	2.51	2.33	1.97	1.68	1.15	0.95	0.90	0.00	0.38	0.79
	H2C1R	7.36	6.58	4.04	4.48	5.62	4.31	3.45	3.72	3.17	2.66	2.45	2.10	1.79	1.30	1.06	0.89	0.37	0.00	0.43
	G6PO	7.32	6.61	4.24	4.51	5.58	4.39	3.61	3.75	3.25	2.80	2.52	2.21	1.91	1.44	1.19	0.97	0.68	0.40	0.00

UN, uncle–nephew; HS, half-sib; GPO, grandparent–grandoffspring; GUGN, great-uncle–great-nephew; C, cousin; HUN, half-uncle–nephew; G2PO, great-grandparent–grandoffspring; C1R, cousin once removed; HC, half-cousin; G3PO, great-great-grandparent–grandoffspring; 2C, second cousin; HC1R, half-cousin once removed; G4PO, great-great-great-grandparent–grandoffspring; 2C1R, second cousin once removed; H2C, half second cousin; G5PO, great-great-great-great-grandparent–grandoffspring; 3C, third cousin; H2C1R, half second cousin once removed; G6PO, great-great-great-great-great-grandparent–grandoffspring.

**Appendix Table A7:** E(*λ*)/SD(*λ*) as Tables A1 and A4 using information only on numbers of shared segments

*R*		1/4			1/8				1/16			1/32			1/64			1/128		
	**R**	UN	HS	GPO	GUGN	C	HUN	G2PO	C1R	HC	G3PO	2C	HC1R	G4PO	2C1R	H2C	G5PO	3C	H2C1R	G6PO
1/4	UN	0.00	0.91	2.99	2.38	1.69	2.67	3.57	3.82	4.36	4.79	5.54	5.88	6.07	7.01	7.23	7.31	8.22	8.39	8.45
	HS	0.96	0.00	2.15	1.83	1.10	2.05	2.89	3.22	3.78	4.20	4.92	5.29	5.51	6.35	6.61	6.72	7.58	7.76	7.82
	GPO	3.54	2.43	0.00	1.90	2.03	1.42	1.25	1.89	2.16	2.48	3.01	3.32	3.62	4.17	4.41	4.63	5.17	5.35	5.49
1/8	GUGN	1.86	1.41	1.53	0.00	0.54	0.46	1.36	1.10	1.54	2.03	2.27	2.55	2.81	3.20	3.38	3.54	3.94	4.06	4.17
	C	1.44	0.92	1.60	0.58	0.00	0.87	1.79	1.73	2.20	2.67	3.04	3.34	3.61	4.10	4.30	4.47	4.97	5.11	5.21
	HUN	2.28	1.65	1.23	0.50	0.86	0.00	0.97	0.85	1.28	1.75	2.05	2.33	2.60	2.99	3.18	3.36	3.75	3.88	3.99
	G2PO	3.52	2.68	1.10	1.69	2.04	1.12	0.00	0.79	0.79	1.03	1.43	1.67	1.92	2.31	2.48	2.66	3.03	3.16	3.27
1/16	C1R	3.06	2.47	1.64	1.11	1.61	0.82	0.70	0.00	0.44	0.94	1.13	1.39	1.66	1.98	2.14	2.31	2.63	2.74	2.85
	HC	3.63	2.98	1.86	1.59	2.10	1.25	0.76	0.45	0.00	0.53	0.71	0.96	1.23	1.55	1.71	1.88	2.19	2.30	2.40
	G3PO	4.47	3.69	2.11	2.30	2.81	1.86	1.00	1.07	0.59	0.00	0.46	0.62	0.83	1.18	1.34	1.50	1.81	1.92	2.02
1/32	2C	4.61	3.93	2.58	2.34	2.91	2.02	1.39	1.15	0.72	0.45	0.00	0.27	0.56	0.83	0.99	1.16	1.44	1.54	1.64
	HC1R	5.10	4.37	2.85	2.69	3.29	2.35	1.64	1.46	0.99	0.62	0.27	0.00	0.31	0.58	0.74	0.91	1.19	1.30	1.40
	G4PO	5.78	4.94	3.16	3.18	3.82	2.78	1.92	1.85	1.35	0.85	0.62	0.34	0.00	0.37	0.51	0.66	0.97	1.07	1.17
1/64	2C1R	6.31	5.48	3.70	3.49	4.19	3.14	2.35	2.15	1.66	1.23	0.88	0.60	0.38	0.00	0.17	0.35	0.63	0.73	0.83
	H2C	6.77	5.89	3.97	3.79	4.53	3.42	2.57	2.38	1.87	1.41	1.07	0.78	0.53	0.18	0.00	0.19	0.47	0.57	0.68
	G5PO	7.39	6.42	4.29	4.19	4.97	3.78	2.83	2.69	2.14	1.63	1.31	1.00	0.70	0.39	0.21	0.00	0.32	0.42	0.52
1/128	3C	8.26	7.24	4.96	4.68	5.56	4.29	3.33	3.10	2.53	2.03	1.64	1.34	1.05	0.67	0.49	0.33	0.00	0.11	0.23
	H2C1R	8.71	7.64	5.23	4.94	5.87	4.53	3.54	3.30	2.71	2.19	1.80	1.48	1.18	0.80	0.61	0.44	0.12	0.00	0.12
	G6PO	9.30	8.15	5.54	5.30	6.28	4.85	3.77	3.56	2.94	2.37	1.98	1.65	1.32	0.94	0.75	0.55	0.24	0.13	0.00

UN, uncle–nephew; HS, half-sib; GPO, grandparent–grandoffspring; GUGN, great-uncle–great-nephew; C, cousin; HUN, half-uncle–nephew; G2PO, great-grandparent–grandoffspring; C1R, cousin once removed; HC, half-cousin; G3PO, great-great-grandparent–grandoffspring; 2C, second cousin; HC1R, half-cousin once removed; G4PO, great-great-great-grandparent–grandoffspring; 2C1R, second cousin once removed; H2C, half second cousin; G5PO, great-great-great-great-grandparent–grandoffspring; 3C, third cousin; H2C1R, half second cousin once removed; G6PO, great-great-great-great-great-grandparent–grandoffspring.

**Appendix Table A8:** E(*λ*)/SD(*λ*) as Tables A2 and A5 using information on numbers and position of shared segments

*R*		1/4			1/8				1/16			1/32			1/64			1/128		
	**R**	UN	HS	GPO	GUGN	C	HUN	G2PO	C1R	HC	G3PO	2C	HC1R	G4PO	2C1R	H2C	G5PO	3C	H2C1R	G6PO
1/4	UN	0.00	0.97	3.27	2.47	2.04	2.78	3.59	3.84	4.38	4.90	5.24	5.66	6.10	6.37	6.68	7.12	7.24	7.44	7.95
	HS	1.02	0.00	2.37	2.05	1.80	2.20	2.94	3.25	3.69	4.27	4.45	4.84	5.32	5.51	5.79	6.18	6.34	6.60	6.92
	GPO	3.83	2.66	0.00	2.55	2.76	2.24	1.78	2.80	2.77	2.79	3.29	3.38	3.47	3.92	4.00	4.09	4.52	4.67	4.65
1/8	GUGN	2.12	1.95	2.64	0.00	0.60	0.51	1.54	1.24	1.57	2.03	2.34	2.57	2.83	3.19	3.36	3.55	3.84	3.96	4.13
	C	1.95	1.94	2.93	0.64	0.00	0.99	2.06	1.75	2.20	2.69	3.02	3.32	3.60	3.99	4.21	4.43	4.75	4.90	5.12
	HUN	2.42	2.00	2.44	0.55	0.99	0.00	1.12	1.11	1.35	1.76	2.13	2.36	2.62	2.96	3.14	3.33	3.61	3.74	3.91
	G2PO	3.54	2.74	1.94	1.84	2.31	1.26	0.00	1.36	1.18	1.16	1.76	1.85	1.98	2.38	2.49	2.62	2.93	3.06	3.14
1/16	C1R	3.56	3.26	3.52	1.31	1.67	1.21	1.46	0.00	0.51	1.08	1.14	1.39	1.66	1.98	2.14	2.31	2.62	2.72	2.84
	HC	3.94	3.48	3.49	1.64	2.11	1.37	1.32	0.53	0.00	0.61	0.81	0.99	1.24	1.59	1.73	1.88	2.18	2.28	2.39
	G3PO	4.62	3.96	3.29	2.30	2.83	1.87	1.24	1.23	0.67	0.00	0.78	0.77	0.87	1.31	1.40	1.53	1.84	1.93	2.02
1/32	2C	5.17	4.70	4.60	2.57	3.02	2.35	2.19	1.19	0.88	0.86	0.00	0.31	0.63	0.83	0.99	1.16	1.44	1.54	1.64
	HC1R	5.52	4.96	4.54	2.81	3.34	2.54	2.20	1.46	1.04	0.85	0.32	0.00	0.35	0.62	0.75	0.91	1.21	1.30	1.40
	G4PO	6.08	5.38	4.49	3.23	3.83	2.87	2.26	1.86	1.35	0.93	0.71	0.38	0.00	0.52	0.57	0.68	1.02	1.10	1.18
1/64	2C1R	6.93	6.30	5.64	3.75	4.34	3.48	3.05	2.20	1.80	1.55	0.89	0.67	0.58	0.00	0.20	0.40	0.63	0.73	0.83
	H2C	7.30	6.59	5.67	3.97	4.62	3.67	3.12	2.41	1.96	1.63	1.07	0.81	0.63	0.20	0.00	0.22	0.49	0.58	0.67
	G5PO	7.82	7.00	5.73	4.30	5.02	3.94	3.24	2.70	2.18	1.75	1.31	1.01	0.73	0.44	0.23	0.00	0.38	0.44	0.52
1/128	3C	8.98	8.14	6.88	4.97	5.74	4.64	3.99	3.18	2.69	2.31	1.67	1.40	1.19	0.68	0.53	0.42	0.00	0.13	0.25
	H2C1R	9.36	8.46	6.99	5.18	6.00	4.83	4.10	3.35	2.82	2.40	1.81	1.52	1.27	0.80	0.63	0.48	0.13	0.00	0.14
	G6PO	9.88	8.87	7.11	5.47	6.37	5.08	4.24	3.58	3.01	2.52	1.98	1.66	1.38	0.95	0.75	0.57	0.27	0.15	0.00

UN, uncle–nephew; HS, half-sib; GPO, grandparent–grandoffspring; GUGN, great-uncle–great-nephew; C, cousin; HUN, half-uncle–nephew; G2PO, great-grandparent–grandoffspring; C1R, cousin once removed; HC, half-cousin; G3PO, great-great-grandparent–grandoffspring; 2C, second cousin; HC1R, half-cousin once removed; G4PO, great-great-great-grandparent–grandoffspring; 2C1R, second cousin once removed; H2C, half second cousin; G5PO, great-great-great-great-grandparent–grandoffspring; 3C, third cousin; H2C1R, half second cousin once removed; G6PO, great-great-great-great-great-grandparent–grandoffspring.

**Appendix Table A9:** E(*λ*)/SD(*λ*) as Tables A3 and A6 using information on numbers, position, and length of shared segments

*R*		1/4			1/8				1/16			1/32			1/64			1/128		
	**R**	UN	HS	GPO	GUGN	C	HUN	G2PO	C1R	HC	G3PO	2C	HC1R	G4PO	2C1R	H2C	G5PO	3C	H2C1R	G6PO
1/4	UN	0.00	0.98	3.28	2.95	2.75	3.18	3.76	4.69	5.04	5.40	6.23	6.44	6.86	7.71	7.71	8.03	9.06	9.05	9.19
	HS	1.03	0.00	2.37	2.62	2.62	2.66	3.17	4.24	4.37	4.77	5.60	5.67	6.02	6.92	6.84	7.03	8.11	8.07	8.09
	GPO	3.84	2.66	0.00	3.13	3.47	2.73	2.09	3.96	3.64	3.39	4.67	4.47	4.31	5.42	5.25	5.08	6.05	5.98	5.79
1/8	GUGN	2.64	2.63	3.27	0.00	0.70	0.56	1.68	1.50	1.64	2.03	2.57	2.68	2.87	3.41	3.48	3.62	4.09	4.14	4.23
	C	2.80	2.94	3.67	0.73	0.00	1.13	2.28	1.86	2.22	2.69	3.15	3.39	3.62	4.12	4.29	4.48	4.93	5.06	5.20
	HUN	2.82	2.51	3.02	0.60	1.13	0.00	1.22	1.52	1.49	1.77	2.47	2.52	2.69	3.26	3.30	3.43	3.91	3.96	4.03
	G2PO	3.72	2.97	2.30	1.92	2.50	1.34	0.00	1.94	1.57	1.33	2.39	2.26	2.21	2.94	2.87	2.86	3.43	3.41	3.40
1/16	C1R	4.79	4.75	4.97	1.71	1.85	1.80	2.20	0.00	0.65	1.32	1.19	1.39	1.68	2.03	2.15	2.30	2.66	2.75	2.84
	HC	4.76	4.46	4.77	1.75	2.14	1.60	1.86	0.66	0.00	0.73	1.03	1.05	1.23	1.73	1.79	1.90	2.29	2.35	2.42
	G3PO	5.18	4.60	4.19	2.31	2.85	1.90	1.49	1.43	0.78	0.00	1.21	1.02	0.95	1.63	1.59	1.61	2.07	2.07	2.10
1/32	2C	6.82	6.62	6.99	3.11	3.35	3.10	3.34	1.28	1.23	1.46	0.00	0.42	0.83	0.86	0.99	1.17	1.46	1.55	1.64
	HC1R	6.79	6.40	6.57	3.09	3.49	2.96	3.05	1.47	1.16	1.24	0.43	0.00	0.46	0.74	0.78	0.91	1.28	1.33	1.40
	G4PO	7.07	6.47	5.97	3.33	3.87	3.06	2.76	1.90	1.37	1.07	0.88	0.48	0.00	0.81	0.72	0.72	1.18	1.19	1.22
1/64	2C1R	9.05	8.65	8.87	4.44	4.82	4.37	4.50	2.37	2.20	2.29	0.95	0.89	1.01	0.00	0.28	0.54	0.64	0.73	0.84
	H2C	9.06	8.52	8.32	4.42	4.93	4.27	4.19	2.48	2.17	2.11	1.09	0.88	0.87	0.29	0.00	0.30	0.55	0.59	0.67
	G5PO	9.29	8.57	7.78	4.56	5.19	4.31	3.96	2.72	2.26	2.01	1.35	1.02	0.82	0.57	0.31	0.00	0.55	0.52	0.54
1/128	3C	11.56	10.93	10.59	5.79	6.37	5.65	5.56	3.41	3.13	3.08	1.76	1.62	1.62	0.72	0.65	0.70	0.00	0.19	0.35
	H2C1R	11.63	10.88	10.12	5.79	6.47	5.60	5.33	3.49	3.12	2.96	1.85	1.63	1.54	0.82	0.67	0.63	0.19	0.00	0.20
	G6PO	11.86	10.96	9.69	5.90	6.68	5.63	5.17	3.65	3.18	2.89	2.00	1.71	1.52	0.98	0.77	0.62	0.37	0.21	0.00

UN, uncle–nephew; HS, half-sib; GPO, grandparent–grandoffspring; GUGN, great-uncle–great-nephew; C, cousin; HUN, half-uncle–nephew; G2PO, great-grandparent–grandoffspring; C1R, cousin once removed; HC, half-cousin; G3PO, great-great-grandparent–grandoffspring; 2C, second cousin; HC1R, half-cousin once removed; G4PO, great-great-great-grandparent–grandoffspring; 2C1R, second cousin once removed; H2C, half second cousin; G5PO, great-great-great-great-grandparent–grandoffspring; 3C, third cousin; H2C1R, half second cousin once removed; G6PO, great-great-great-great-great-grandparent–grandoffspring.

**Appendix Table A10:** E(*λ*)/SD(*λ*) evaluated using a model of 22 chromosomes, each of length 1.632 M, using information as Table A9 on numbers, position, and length of segments

*R*		1/4			1/8				1/16			1/32			1/64			1/128		
	**R**	UN	HS	GPO	GUGN	C	HUN	G2PO	C1R	HC	G3PO	2C	HC1R	G4PO	2C1R	H2C	G5PO	3C	H2C1R	G6PO
1/4	UN	0.00	0.97	3.25	2.97	2.78	3.19	3.75	4.70	5.04	5.35	6.18	6.43	6.80	7.46	7.67	7.95	8.79	8.75	8.98
	HS	1.03	0.00	2.37	2.65	2.65	2.67	3.17	4.26	4.39	4.77	5.57	5.63	6.01	6.68	6.78	7.00	7.92	7.82	7.86
	GPO	3.83	2.66	0.00	3.15	3.47	2.74	2.09	3.92	3.62	3.39	4.64	4.35	4.31	5.33	5.15	4.98	6.00	5.85	5.65
1/8	GUGN	2.66	2.65	3.25	0.00	0.72	0.56	1.68	1.51	1.64	2.00	2.56	2.69	2.85	3.37	3.48	3.60	4.04	4.10	4.20
	C	2.84	2.96	3.63	0.75	0.00	1.13	2.28	1.88	2.24	2.68	3.15	3.42	3.62	4.12	4.33	4.50	4.91	5.04	5.20
	HUN	2.83	2.53	3.01	0.59	1.14	0.00	1.22	1.54	1.50	1.76	2.47	2.53	2.69	3.23	3.31	3.43	3.88	3.93	4.00
	G2PO	3.72	2.97	2.29	1.92	2.50	1.33	0.00	1.94	1.57	1.32	2.39	2.25	2.21	2.89	2.85	2.85	3.40	3.38	3.35
1/16	C1R	4.83	4.81	4.94	1.72	1.87	1.82	2.21	0.00	0.65	1.32	1.19	1.40	1.67	2.03	2.17	2.30	2.66	2.76	2.85
	HC	4.79	4.50	4.77	1.74	2.16	1.61	1.87	0.67	0.00	0.72	1.04	1.06	1.22	1.73	1.80	1.89	2.29	2.35	2.42
	G3PO	5.17	4.59	4.17	2.30	2.85	1.88	1.47	1.43	0.77	0.00	1.23	1.04	0.96	1.64	1.61	1.62	2.08	2.09	2.11
1/32	2C	6.85	6.67	6.97	3.11	3.37	3.11	3.35	1.27	1.23	1.48	0.00	0.41	0.83	0.86	0.99	1.16	1.47	1.55	1.64
	HC1R	6.83	6.46	6.57	3.09	3.52	2.97	3.06	1.47	1.16	1.26	0.42	0.00	0.45	0.74	0.78	0.89	1.28	1.34	1.40
	G4PO	7.05	6.46	5.96	3.31	3.87	3.04	2.74	1.89	1.34	1.07	0.88	0.47	0.00	0.81	0.73	0.72	1.19	1.21	1.23
1/64	2C1R	9.03	8.67	8.84	4.42	4.82	4.36	4.49	2.36	2.19	2.30	0.93	0.87	1.01	0.00	0.27	0.53	0.64	0.73	0.84
	H2C	9.09	8.57	8.34	4.41	4.95	4.28	4.20	2.48	2.16	2.13	1.08	0.87	0.88	0.28	0.00	0.28	0.54	0.59	0.67
	G5PO	9.26	8.56	7.78	4.52	5.17	4.29	3.94	2.69	2.23	2.01	1.33	0.99	0.80	0.56	0.29	0.00	0.56	0.54	0.55
1/128	3C	11.57	10.98	10.62	5.78	6.38	5.65	5.56	3.40	3.12	3.10	1.75	1.60	1.62	0.71	0.63	0.70	0.00	0.18	0.35
	H2C1R	11.71	10.98	10.18	5.81	6.52	5.63	5.36	3.50	3.13	3.00	1.85	1.63	1.56	0.82	0.66	0.64	0.18	0.00	0.19
	G6PO	11.87	10.99	9.71	5.88	6.69	5.63	5.17	3.64	3.17	2.91	1.99	1.69	1.53	0.97	0.75	0.62	0.36	0.19	0.00

UN, uncle–nephew; HS, half-sib; GPO, grandparent–grandoffspring; GUGN, great-uncle–great-nephew; C, cousin; HUN, half-uncle–nephew; G2PO, great-grandparent–grandoffspring; C1R, cousin once removed; HC, half-cousin; G3PO, great-great-grandparent–grandoffspring; 2C, second cousin; HC1R, half-cousin once removed; G4PO, great-great-great-grandparent–grandoffspring; 2C1R, second cousin once removed; H2C, half second cousin; G5PO, great-great-great-great-grandparent–grandoffspring; 3C, third cousin; H2C1R, half second cousin once removed; G6PO, great-great-great-great-great-grandparent–grandoffspring.

**Appendix Table A11:** Skew coefficient of log likelihood ratio for a subset of relationships for the model in Table A10 of 22 chromosomes, each of 1.632 M, using information on numbers, positions, and lengths of shared segments

*R*		1/4			1/8			1/32			1/128		
	**R**	UN	HS	GPO	C	HUN	G2PO	2C	HC1R	G4PO	3C	H2C1R	G6PO
1/4	UN		0.15	0.11	−0.03	−0.21	−0.01	−0.09	−0.13	−0.17	−0.17	−0.16	−0.17
	HS	−0.16		0.12	0.05	−0.13	−0.13	−0.06	−0.07	−0.16	−0.16	−0.15	−0.14
	GPO	−0.20	−0.18		0.09	0.09	0.04	0.02	0.06	0.02	−0.08	−0.06	−0.03
1/8	C	−0.09	−0.20	0.08		−0.02	0.10	−0.05	−0.13	−0.09	−0.06	−0.08	−0.11
	HUN	0.02	−0.01	−0.02	−0.06		0.26	0.15	0.08	0.02	0.05	0.04	0.02
	G2PO	−0.02	−0.01	−0.23	−0.08	−0.14		0.23	0.25	0.16	0.11	0.12	0.12
1/32	2C	−0.25	−0.28	−0.42	−0.20	−0.38	−0.46		0.19	0.32	0.21	0.18	0.18
	HC1R	−0.23	−0.24	−0.45	−0.18	−0.27	−0.46	−0.08		0.45	0.31	0.24	0.20
	G4PO	−0.26	−0.27	−0.43	−0.22	−0.27	−0.38	0.02	−0.10		0.47	0.40	0.33
1/128	3C	−0.51	−0.53	−0.78	−0.48	−0.56	−0.72	−0.48	−0.60	−0.85		0.61	0.59
	H2C1R	−0.57	−0.58	−0.76	−0.53	−0.59	−0.69	−0.51	−0.57	−0.71	−0.15		0.65
	G6PO	−0.59	−0.60	−0.73	−0.56	−0.60	−0.66	−0.55	−0.56	−0.62	−0.07	0.02	

UN, uncle–nephew; HS, half-sib; GPO, grandparent–grandoffspring; C, cousin; HUN, half-uncle–nephew; G2PO, great-grandparent–grandoffspring; 2C, second cousin; HC1R, half-cousin once removed; G4PO, great-great-great-grandparent–grandoffspring; 3C, third cousin; H2C1R, half second cousin once removed; G6PO, great-great-great-great-great-grandparent–grandoffspring.

**Appendix Table A12:** Kurtosis coefficient of log likelihood ratio, otherwise as Table A11

*R*		1/4			1/8			1/32			1/128		
	**R**	UN	HS	GPO	C	HUN	G2PO	2C	HC1R	G4PO	3C	H2C1R	G6PO
1/4	UN		0.04	0.02	0.02	0.09	0.03	0.02	0.04	0.08	0.04	0.03	0.04
	HS	0.00		0.00	−0.05	0.01	0.07	−0.05	−0.02	0.03	0.00	0.00	0.03
	GPO	0.03	0.03		−0.02	−0.04	−0.05	−0.06	−0.05	−0.05	−0.06	−0.05	−0.05
1/8	C	0.01	0.07	0.11		0.00	0.05	−0.01	−0.02	−0.02	−0.01	−0.01	−0.01
	HUN	0.00	−0.01	0.07	−0.03		0.11	0.02	0.00	0.01	−0.02	−0.01	−0.01
	G2PO	−0.01	−0.01	0.04	0.11	0.15		0.05	0.07	0.01	−0.02	0.00	−0.01
1/32	2C	−0.04	0.00	0.36	−0.08	0.09	0.40		0.08	0.12	−0.07	−0.09	−0.10
	HC1R	−0.02	−0.01	0.21	−0.05	0.01	0.21	0.24		0.53	0.02	−0.01	−0.04
	G4PO	−0.03	−0.03	0.14	−0.06	−0.03	0.08	0.45	0.57		0.17	0.10	0.04
1/128	3C	0.22	0.25	0.72	0.16	0.30	0.59	0.17	0.37	0.87		1.42	0.75
	H2C1R	0.32	0.33	0.67	0.27	0.35	0.52	0.26	0.31	0.54	1.64		1.55
	G6PO	0.32	0.32	0.53	0.28	0.32	0.42	0.31	0.29	0.35	1.13	2.49	

UN, uncle–nephew; HS, half-sib; GPO, grandparent–grandoffspring; C, cousin; HUN, half-uncle–nephew; G2PO, great-grandparent–grandoffspring; 2C, second cousin; HC1R, half-cousin once removed; G4PO, great-great-great-grandparent–grandoffspring; 3C, third cousin; H2C1R, half second cousin once removed; G6PO, great-great-great-great-great-grandparent–grandoffspring.

**Appendix Table A13:** Influence on E(*λ*)/SD(*λ*) of chromosome number *vs.* length, (72 × 0.5 M or 12 × 3M) for total 36 M, using information only on number of shared segments (*n*_S_) or using their numbers, position, and length *n_s_*, *p_s_*, and *t_s_*

		Analysis using only *n_s_*				Analysis using *n_s_*, *p_s_*, and *t_s_*			
*R*		1/4		1/32		1/4		1/32	
	**R**	HS		HC1R		HS		HC1R	
		Number of chromosomes							
		72	12	72	12	72	12	72	12
		E(*λ*)/SD(*λ*)							
1/4	UN	0.82	0.94	7.65	5.11	0.97	0.98	7.69	6.35
	HS	0.00	0.00	6.84	4.52	0.00	0.00	6.97	5.38
	GPO	2.07	2.53	5.00	2.77	2.68	2.66	5.93	4.34
1/8	C	2.01	1.42	3.86	3.20	3.60	2.60	3.89	3.28
	HUN	2.73	1.29	3.06	2.14	3.27	2.26	3.22	2.34
	G2PO	3.57	2.41	2.45	1.45	3.65	2.77	2.95	2.08
1/32	2C	6.40	3.17	0.27	0.28	8.64	5.86	0.47	0.41
	HC1R	6.89	3.61	0.00	0.00	8.42	5.75	0.00	0.00
	G4PO	7.33	4.17	0.29	0.32	8.32	5.85	0.50	0.45
1/128	3C	11.04	6.10	1.71	1.22	14.36	9.75	2.01	1.48
	H2C1R	11.58	6.47	1.88	1.36	14.38	9.77	2.05	1.50
	G6PO	11.92	6.98	1.99	1.54	14.14	9.94	2.07	1.60

HS, half-sib; HC1R, half-cousin once removed; UN, uncle–nephew; GPO, grandparent–grandoffspring; C, cousin; HUN, half-uncle–nephew; G2PO, great-grandparent–grandoffspring; 2C, second cousin; G4PO, great-great-great-grandparent–grandoffspring; 3C, third cousin; H2C1R, half second cousin once removed; G6PO, great-great-great-great-great-grandparent–grandoffspring.

## References

[bib1] Abecasis G. R. Cherny S. S. Cookson W. O. Cardon L. R., 2002 Merlin - rapid analysis of dense genetic maps using sparse gene flow trees. Nat. Genet. 30: 97–101 11731797 10.1038/ng786

[bib2] Blouin M. S., 2003 DNA-based methods for pedigree reconstruction and kinship analysis in natural populations. Trends Ecol. Evol. 18: 503–511

[bib3] Browning B. L. Browning S. R., 2013 Improving the accuracy and efficiency of identity-by-descent detection in population data. Genetics 194: 459–471 23535385 10.1534/genetics.113.150029 PMC3664855

[bib4] Browning S. R. Browning B. L., 2011 A fast, powerful method for detecting identity by descent. Am. J. Hum. Genet. 88: 173–182 21310274 10.1016/j.ajhg.2011.01.010 PMC3035716

[bib5] Browning S. R. Browning B. L., 2012 Identity by descent between distant relatives: detection and applications. Annu. Rev. Genet. 46: 617–633 22994355 10.1146/annurev-genet-110711-155534

[bib6] Burnham K. P. Anderson D. R., 2001 Kullback–Leibler information as a basis for strong inference in ecological studies. Wildl. Res. 28: 111–119

[bib7] Donnelly K. P., 1983 The probability that related individuals share some section of the genome identical by descent. Theor. Popul. Biol. 23: 34–64 6857549 10.1016/0040-5809(83)90004-7

[bib8] Fisher R. A., 1949 The theory of inbreeding, Oliver and Boyd, London

[bib9] Fisher R. A., 1954 A fuller theory of ‘Junctions’ in inbreeding. Heredity 8: 187–197

[bib10] Epstein E. Duren W. L. Boenkhe M., 2000 Improved inference of relationships for pairs of individuals. Am. J. Hum. Genet. 67: 1219–1231 11032786 10.1016/s0002-9297(07)62952-8 PMC1288564

[bib11] Hill W. G. Weir B. S., 2011 Variation in actual relationship as a consequence of Mendelian sampling and linkage. Genet. Res. 93: 47–74 10.1017/S0016672310000480 PMC3070763 21226974

[bib12] Huff C. D. Witherspoon D. J. Simonson T. S. Xing J. C. Wa W. S., 2011 Maximum-likelihood estimation of recent shared ancestry (ERSA). Genome Res. 21: 768–774 21324875 10.1101/gr.115972.110 PMC3083094

[bib13] Kong X. Murphy K. Raj T. He C. White P. S., 2004 A combined physical-linkage map of the human genome. Am. J. Hum. Genet. 75: 1143–1148 15486828 10.1086/426405 PMC1182151

[bib14] Kullback S. Leibler R. A., 1951 On information and sufficiency. Ann. Math. Stat. 22: 79–86

[bib15] Kyriazopoulou-Panagiotopoulou S. Haghighi D. K. Aerni S. J. Sundquist A. Bercovici S., 2011 Reconstruction of genealogical relationships with applications to Phase III of HapMap. Bioinformatics 27: I333–I341 21685089 10.1093/bioinformatics/btr243 PMC3117348

[bib16] Mardia K. V. Kent J. T. Bibby J. M., 1979 Multivariate Analysis, Academic Press, London

[bib17] Marshall T. C. Slate J. Kruuk L. E. B. Pemberton J. M., 1998 Statistical confidence for likelihood-based paternity inference in natural populations. Mol. Ecol. 7: 639–655 9633105 10.1046/j.1365-294x.1998.00374.x

[bib18] McPeek M. S. Sun L., 2000 Statistical tests for detection of misspecified relationships by use of genome-screen data. Am. J. Hum. Genet. 66: 1076–1094 10712219 10.1086/302800 PMC1288143

[bib19] Pemberton J. M., 2008 Wild pedigrees: the way forward. Proc. Biol. Sci. 275: 613–621 18211868 10.1098/rspb.2007.1531 PMC2386891

[bib20] Purcell S. Neale B. Todd-Brown K. Thomas L. Ferreira M. A. R., 2007 PLINK: a toolset for whole-genome association and population-based linkage analysis. Am. J. Hum. Genet. 81: 559–575 17701901 10.1086/519795 PMC1950838

[bib21] Roberson E. D. O. Pevsner J., 2009 Visualization of shared genomic regions and meiotic recombination in high-density SNP data. PLoS ONE 4: e6711 19696932 10.1371/journal.pone.0006711 PMC2725774

[bib22] Rohlfs R. V. Fullerton S. M. Weir B. S., 2012 Familial identification: population structure and relationship distinguishability. PLoS Genet. 8: e1002469 22346758 10.1371/journal.pgen.1002469 PMC3276546

[bib23] Stam P., 1980 The distribution of the fraction of the genome identical by descent in finite populations. Genet. Res. 35: 131–155

[bib24] Thomas A. Skolnick M. H. Lewis C. M., 1994 Genomic mismatch scanning in pedigrees. IMA J. Math. Appl. Med. Biol. 11: 1–16 8057038 10.1093/imammb/11.1.1

[bib25] Wang J., 2011 COANCESTRY: A program for simulating, estimating and analysing relatedness and inbreeding coefficients. Mol. Ecol. Resources 11: 141–145 10.1111/j.1755-0998.2010.02885.x 21429111

[bib26] Weir B. S., 1996 Genetic data analysis. II, Sinauer, Sunderland, MA

