# Supplementary material for: Identification of Pedigree Relationship from Genome Sharing
Source: G3 (Bethesda). 2013 Sep 1;3(9):1553–71. doi: 10.1534/g3.113.007500 (PMC3755916; doi:10.1534/g3.113.007500)
Supplement: Supporting Information [file supp_3_9_1553__index.html]

Identification of Pedigree Relationship from Genome Sharing — Supporting Information 

# Identification of Pedigree Relationship from Genome Sharing

## Supporting Information for Hill and White, 2013

**Files in this Data Supplement:**

- File S1 - Sets of simulated data as used in the analysis (.zip, 110 MB)
